# ZSWIM8 is a myogenic protein that partly prevents C2C12 differentiation

**DOI:** 10.1038/s41598-021-00306-6

**Published:** 2021-10-22

**Authors:** Fumihiko Okumura, Nodoka Oki, Yuha Fujiki, Rio Ikuta, Kana Osaki, Shun Hamada, Kunio Nakatsukasa, Naoki Hisamoto, Taichi Hara, Takumi Kamura

**Affiliations:** 1 Department of Food and Health Sciences, International College of Arts and Sciences, Fukuoka Women’s University, Fukuoka, 813-8582 Japan; 2 Graduate School of Natural Sciences, Nagoya City University, Aichi, 467-8501 Japan; 3 Division of Biological Science, Graduate School of Science, Nagoya University, Aichi, 464-8602 Japan; 4 Faculty of Human Sciences, Waseda University, Saitama, 359-1192 Japan

**Keywords:** Proteolysis, Cell signalling, Post-translational modifications, Proteolysis, Post-translational modifications, Proteolysis, Stem-cell differentiation

## Abstract

Cell adhesion molecule-related/downregulated by oncogenes (Cdon) is a cell-surface receptor that mediates cell–cell interactions and positively regulates myogenesis. The cytoplasmic region of Cdon interacts with other proteins to form a Cdon/JLP/Bnip-2/CDC42 complex that activates p38 mitogen-activated protein kinase (MAPK) and induces myogenesis. However, Cdon complex may include other proteins during myogenesis. In this study, we found that Cullin 2-interacting protein zinc finger SWIM type containing 8 (ZSWIM8) ubiquitin ligase is induced during C2C12 differentiation and is included in the Cdon complex. We knocked-down *Zswim8* in C2C12 cells to determine the effect of ZSWIM8 on differentiation. However, we detected neither ZSWIM8-dependent ubiquitination nor the degradation of Bnip2, Cdon, or JLP. In contrast, ZSWIM8 knockdown accelerated C2C12 differentiation. These results suggest that ZSWIM8 is a Cdon complex-included myogenic protein that prevents C2C12 differentiation without affecting the stability of Bnip2, Cdon, and JLP.

## Introduction

Satellite cells are muscle stem cells located between the sarcolemma and the basal lamina of skeletal muscles. Upon muscle tissue damage, some satellite cells exit a quiescent state to expand as myoblasts. Myoblasts differentiate and fuse with each other to form myofibers^1,2^. p38 mitogen-activated protein kinase (MAPK) exists as four isoforms (α, β, γ, and δ), of which p38α plays an essential role in myoblast differentiation^3^. p38α/β MAPK is recognized as the master kinase for the proliferation-to-differentiation transition of satellite cells^2,4–7^. Moreover, p38α/β MAPK regulates the expression and/or activity of many proteins involved in the transcriptional and epigenetic regulation of myogenesis^2^.

The ubiquitin–proteasome system regulates various cellular processes such as cell-cycle progression, gene transcription, and signal transduction via degradation of ubiquitinated proteins by a proteasome^8^. The covalent attachment of ubiquitin to a substrate is attributed to ubiquitin-activating enzyme (E1), ubiquitin-conjugating enzyme (E2), and ubiquitin ligase (E3). E3 is primarily responsible for substrate recognition^9^. The Elongin B and C–Cullin (Cul) 2 or Cul5-SOCS box protein family is a member of the largest RING finger E3 superfamily, the Cullin-RING-ligases^10^. The Von Hippel–Lindau (VHL) box consists of a BC box, which recruits an adaptor protein (Elongin B and C) and Cul2 box, which binds to Cul2, a scaffold protein that assembles multiple proteins such as small RING finger protein Rbx1, Elongin B and C, and a substrate-targeting protein (VHL box protein) into complexes ^10–12^. Zinc finger SWIM type containing 8 (ZSWIM8) contains a VHL box and SWIM domain and is thus recognized as a Cul2-type ubiquitin ligase^10,13^. A SWIM domain is found in bacterial SWI2/SNF2 ATPases and MuDR transposases and is predicted to have DNA-binding and protein–protein interaction functions in different contexts^13^. ZSWIM8 destabilizes the mutant Robo3(I66L) associated with horizontal gaze palsy with progressive scoliosis (HGPPS) but does not destabilize the wild-type Robo3^14^. The Robo receptor family consists of four members (Robo1, Robo2, Robo3, and Robo4) that are single-pass, type I membrane proteins that regulate neurogenesis, angiogenesis, organ development, and cancer progression^15^. To date, mutant Robo3(I66L) is the only known substrate of ZSWIM8 to be ubiquitinated and degraded by a proteasome. Importantly, ubiquitin ligase activity of ZSWIM8 under normal physiological conditions has not been elucidated, and therefore, ZSWIM8 may have functions other than being a ubiquitin ligase.

Cell adhesion molecule-related/downregulated by oncogenes (Cdon) is a Robo-related cell surface protein positively regulating myogenesis^16–18^. Cdon is upregulated during C2C12 myoblast differentiation and accelerates myogenesis^17^. The Cdon intracellular region binds to c-Jun NH2-terminal kinase (JNK)-associated leucine zipper protein (JLP), a scaffold protein for the p38α/β MAPK pathway^19^. Cdon also interacts with BCL2 adenovirus E1B 19 kDa interacting protein 2 (Bnip-2) and cell division cycle 42 (Cdc42), and the Cdon/JLP/Bnip-2/Cdc42 complex activates p38α/β MAPK and induces myogenesis^20^.

Here, we aimed to identify the substrate of the ubiquitin ligase, ZSWIM8, to elucidate its biological function and identified Cdon as a ZSWIM8-interacting protein. However, it does not induce the ubiquitination and degradation of Cdon.

## Results

### C2C12 differentiation induced ZSWIM8

**Figure 1:**
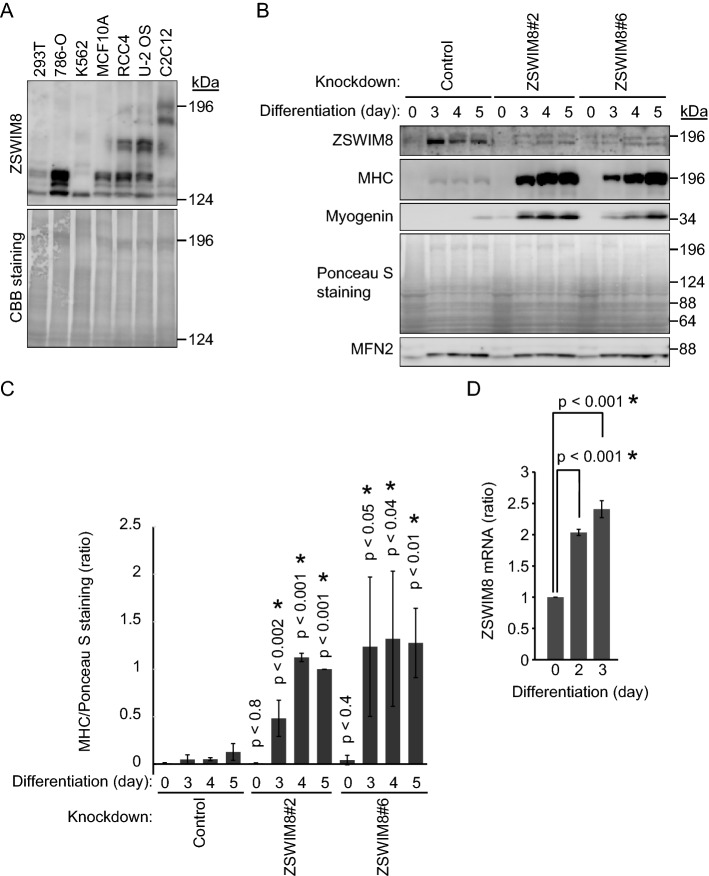
Prevention of C2C12 differentiation by ZSWIM8. (**A**) Expression of ZSWIM8 in C2C12 myoblasts. ZSWIM8 expression in several cell lines was examined. Ponceau S staining rather than a particular protein, such as tubulin or actin, was used as a loading control. The expected ZSWIM8 molecular weight is 197,781 Da. (**B**) Prevention of C2C12 differentiation by ZSWIM8. Control or ZSWIM8-knockdown (#2 and #6) C2C12 cells were differentiated for 3, 4, or 5 days. The cell lysates were subjected to immunoblotting with an anti-ZSWIM8, myosin heavy chain (MHC), or myogenin antibody. Mitofusin 2 (MFN2) and Ponceau S staining were used as loading controls. Representative data of three independent experiments. (**C**) Quantification of MHC expression in (**B**). MHC signals were normalized to that of Ponceau S staining. Expression in ZSWIM8 knockdown#2 cells after 5 days differentiation was set as 1. Data represent the mean ± SD of three independent experiments. (**D**) Induction of *Zswim8* mRNA during C2C12 cell differentiation. *Zswim8* mRNA expression in growing or differentiated C2C12 cells was measured using quantitative PCR. Expression in growing cells was set as 1. Data represent the mean ± SD of three independent experiments. Asterisk indicates statistical significance compared to the control sample. The membranes in (**A**) and (**B**) were cut prior to hybridization with antibodies. Full-length blots are presented in Supplementary Fig. 7.

It was not known whether ZSWIM8 is expressed ubiquitously, and therefore, we first examined several available cell lines, such as HEK293T (human embryonic kidney cell), 786-O (human renal cell adenocarcinoma), K562 (human chronic myelogenous leukemia), MCF10A (human fibrocystic disease), RCC4 (human renal clear cell carcinoma), U-2 OS (human osteosarcoma), and C2C12 (mouse myoblast) (Fig. 1A). ZSWIM8 (GenBank Accession number: NM_015037) is encoded by a 5,529-base pair (bp) complementary DNA (cDNA) and its expected protein molecular weight (MW) is approximately 198 kDa. Only C2C12 cells showed a candidate ZSWIM8 signal at the expected MW. C2C12 is a myoblast cell line and is often utilized to study cellular differentiation and cell fusion to form myotubes. We knocked down *Zswim8* in C2C12 cells by targeting two different sequences of *Zswim8* mRNA to exclude off-target effects and established two independent cell lines, Zswim8#2 and #6 (Fig. 1B). Control and ZSWIM8-knockdown cells were induced to terminally differentiate for up to 5 days. Both skeletal myosin heavy chain (MHC) and myogenin were induced by differentiation and utilized as marker proteins. ZSWIM8 knockdown increased the expression of MHC and myogenin compared with that in control cells, indicating that ZSWIM8 prevents the differentiation of C2C12 cells (Fig. 1B and C, and Supplementary Fig. 1). Importantly, the expression of ZSWIM8 was upregulated after 3 days of differentiation, indicating that ZSWIM8 is induced by differentiation (Fig. 1B and Supplementary Fig. 1). Therefore, we examined *Zswim8* mRNA expression during differentiation (Fig. 1D). As expected, it was approximately twofold upregulated 2 days after the induction of differentiation in C2C12 cells. However, control cells differentiated slower than the generally utilized C2C12 cells^20^. Therefore, we established other ZSWIM8 knockdown cell lines utilizing C2C12 cells provided by RIKEN (RCB0987, lot #41) to examine the effect of ZSWIM8 under faster differentiation conditions. These cell lines were used only for experiments, the results of which are presented in Supplementary Fig. 2. ZSWIM8 was induced after 1 day of differentiation, and MHC and myogenin were detectable after 1–2 days of differentiation in the control knockdown cells as in the generally utilized C2C12 cells (Supplementary Fig. 2A). Importantly, ZSWIM8 knockdown increased the expression of MHC and myogenin compared with that in control cells after 2 days of differentiation (Supplementary Fig. 2B). Altogether, these results indicate that ZSWIM8 was induced during C2C12 differentiation while its depletion accelerated differentiation.

### ZSWIM8 knockdown accelerates myotube formation

**Figure 2:**
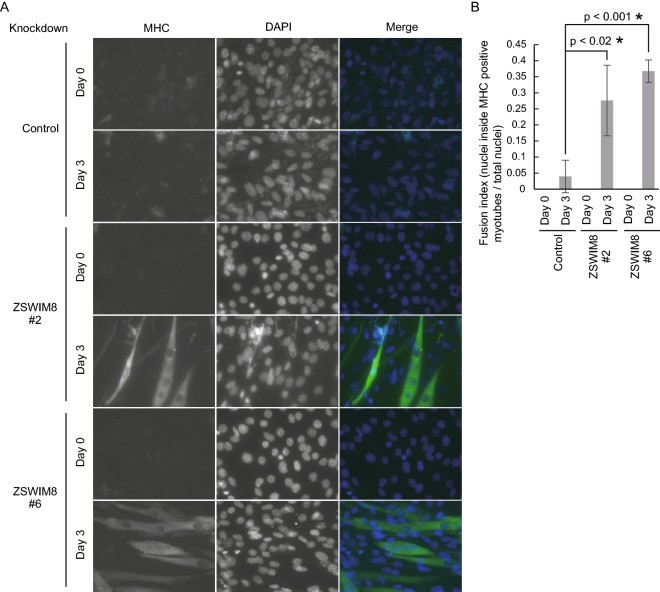
Accelerated myotube formation with ZSWIM8 knockdown. (**A**) Growing (day 0) or differentiated (day 3) control or ZSWIM8-knockdown (#2 or #6) C2C12 cells were immunostained with an anti-myosin heavy chain (MHC) antibody. Nuclei were stained with DAPI. Scale bar, 20 μm. MHC-positive and multinucleated cells were considered myotubes. (**B**) Quantification of fusion indexes of (**A**). The number of nuclei inside MHC-positive myotubes was divided by total nuclei in different fields. Data represent the mean ± SD; n = 150. Asterisk indicates statistical significance compared to the control sample.

Although MHC and myogenin levels were increased in ZSWIM8-knockdown cells compared with that in control cells (Fig. 1B), whether myotube formation was accelerated by ZSWIM8 knockdown during differentiation was unclear. We then examined the morphology of differentiated control or ZSWIM8-knockdown C2C12 cells using an anti-MHC antibody (Fig. 2A). After 3 days of differentiation, ZSWIM8 knockdown increased the fusion index (nuclei inside MHC-positive myotubes/total nuclei) compared to that of control cells (Fig. 2A and B). These data indicate that ZSWIM8 prevents C2C12 myotube formation.

### ZSWIM8 interacts with Cdon

**Figure 3:**
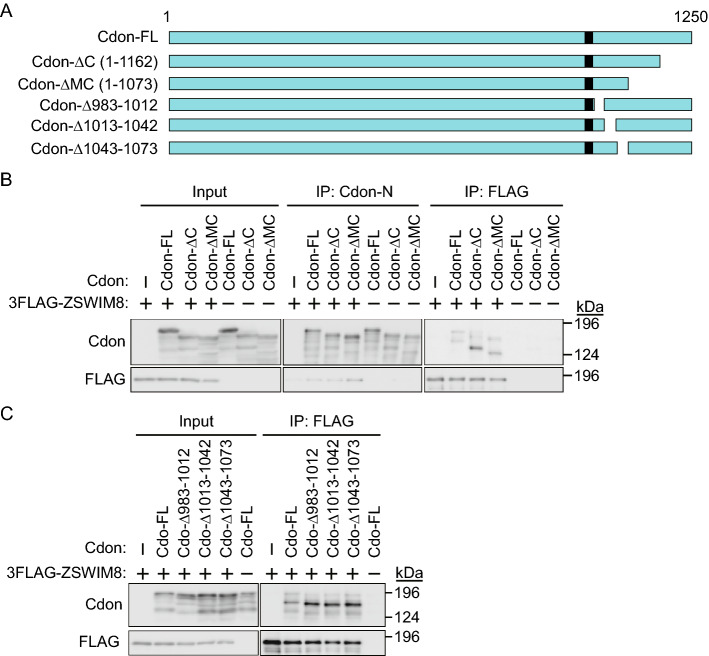
Interaction between ZSWIM8 and Cdon. (**A**) Schematic representation of full-length (FL) or C-terminally-deleted Cdon (ΔC; 1–1162, ΔMC; 1–1073, Δ983–1012, Δ1013–1042, and Δ1043–1073). The black box indicates the transmembrane (TM) region. (**B**) The juxtamembrane region of Cdon is recognized by ZSWIM8. HEK293T cells were transfected with empty plasmid or plasmid encoding non-tagged Cdon (FL, ΔC, or ΔMC) with or without 3 × FLAG-ZSWIM8 and cultured for 2 days. The cell lysates were immunoprecipitated with an anti-Cdon-N or FLAG antibody and immunoblotted with an anti-Cdon-N or FLAG antibody. Representative data of three independent experiments. (**C**) Deletion of the juxtamembrane region of Cdon did not affect the interaction with ZSWIM8. The experiment was performed as in (**B**). The membranes in (**B**) and (**C**) were cut prior to hybridization with antibodies. Full-length blots are presented in Supplementary Fig. 8.

**Figure 4:**
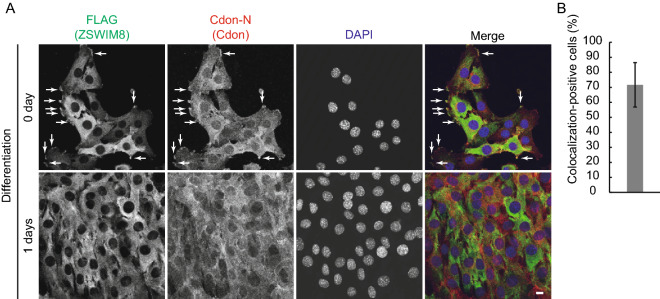
Co-localization of ZSWIM8 and Cdon. (**A**) 3 × FLAG-ZSWIM8-expressing C2C12 cells were immunostained by anti-FLAG and Cdon-N antibodies. Nuclei were stained with DAPI. Scale bar, 10 μm. Representative colocalization in growing cells is indicated by white arrows. Representative data of two independent experiments. (**B**) Quantification of colocalization-positive cells in (**A**). Colocalization-positive cell number was counted and divided by total cell number in four different fields. Data represent the mean ± SD (n = 44).

ZSWIM8 contains a VHL box and is thus recognized as a Cul2-type ubiquitin ligase^10,13^. ZSWIM8 destabilizes mutant Robo3(I66L), associated with HGPPS, but not wild-type Robo3^14^. Cdon is a Robo-related cell surface protein that positively regulates myogenesis^16–18^. Therefore, we speculated that ZSWIM8 might recognize and destabilize Cdon in a ubiquitin proteasome-dependent manner. To confirm the interaction between ZSWIM8 and Cdon, 3 × FLAG-tagged ZSWIM8 (3 × FLAG-ZSWIM8) and full-length Cdon (Cdon-FL) or mutant Cdon (Cdon-ΔC, which lacks amino acids 1163–1250, or Cdon-ΔMC, which lacks amino acids 1074–1250) were expressed in HEK293T cells (Fig. 3A and B). The cell lysates were subjected to immunoprecipitation (IP) with an anti-FLAG antibody, and the immunoprecipitates were analyzed by sodium dodecyl sulfate–polyacrylamide gel electrophoresis (SDS-PAGE) and immunoblotting (IB) with an anti-Cdon-N antibody, which recognizes the external domain of Cdon, or an anti-FLAG antibody. Here, we detected the interaction between ZSWIM8 and Cdon (FL or mutants). Several short Cdon fragments of approximately 130–150 kDa were also co-immunoprecipitated with 3 × FLAG-ZSWIM8 (Fig. 3B). We further confirmed the interaction of ZSWIM8 with three Cdon mutants (Cdon-Δ983-1012, which lacks amino acids 983–1012, Cdon-Δ1013-1042, which lacks amino acids 1013–1042, or Cdon-Δ1043-1073, which lacks amino acids 1043–1073) (Fig. 3A and C). We next examined the cellular localization of Cdon mutants (Supplementary Fig. 3). Cdon and all mutants, except Cdon-Δ983-1012, localized to the cell membrane and cytosol. In contrast, Cdon-Δ983-1012 mainly localized to the perinuclear region. These data suggested that ZSWIM8 recognized multiple regions of Cdon directly, or via other proteins between ZSWIM8 and Cdon in a localization-independent manner. Subsequently, we examined the localization of ZSWIM8 before and during differentiation. As the anti-ZSWIM8 antibody showed a high background in immunofluorescence microscopy, we used C2C12 cells stably expressing 3 × FLAG-ZSWIM8. Here, 3 × FLAG-ZSWIM8 partly co-localized with Cdon in the juxtamembrane region of approximately 70% of the cells (Fig. 4A and B). However, whether they co-localized after 1 day of differentiation was unclear as their localization was diffused in confluent culture after 1 day of differentiation. These data indicate that 3 × FLAG-ZSWIM8 interacts with Cdon at least in the non-differentiated juxtamembrane region.

### ZSWIM8 overexpression prevents C2C12 differentiation

**Figure 5:**
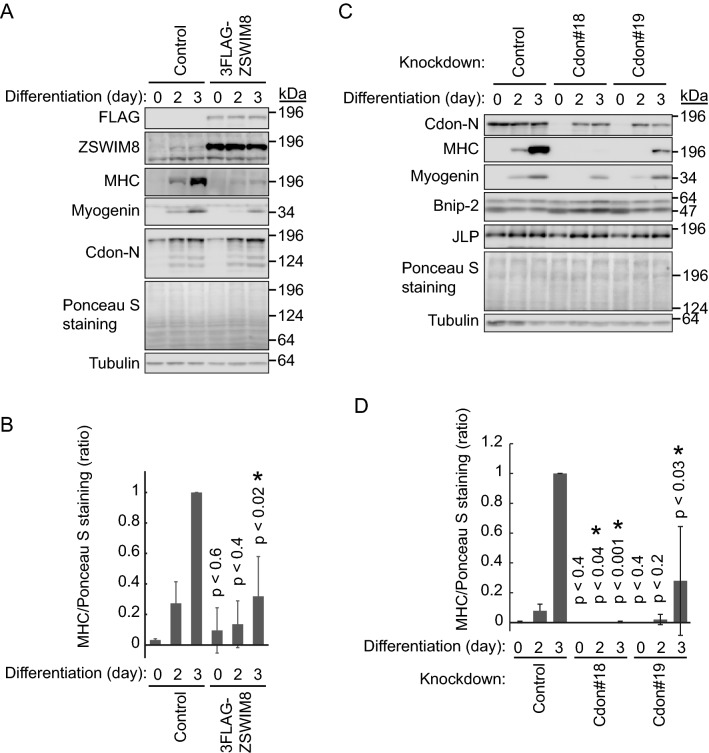
Prevention of C2C12 differentiation by ZSWIM8 overexpression or knockdown of Cdon. (**A**) Prevention of C2C12 differentiation by overexpression of ZSWIM8. Control or 3 × FLAG-ZSWIM8-expressing C2C12 cells were differentiated for 2 or 3 days. The cell lysates were subjected to immunoblotting with an anti-FLAG, ZSWIM8, myosin heavy chain (MHC), myogenin, or Cdon-N antibody. Tubulin and Ponceau S staining were used as loading controls. Representative data of three independent experiments. (**B**) Quantification of MHC expression in (**A**). MHC signals were normalized to that of Ponceau S staining. Expression in control cells after 3 days of differentiation was set as 1. Data represent the mean ± SD of three independent experiments. (**C**) Prevention of C2C12 differentiation by Cdon knockdown. Control or Cdon-knockdown (#18 or 19) C2C12 cells were differentiated for 2 or 3 days. The cell lysates were subjected to immunoblotting with an anti-Cdon-N, MHC, myogenin, Bnip-2, or JLP antibody. Bnip-2 showed doublet bands. Tubulin and Ponceau S staining were used as loading controls. Representative data of three independent experiments. (D) Quantification of MHC expression in (**C**). MHC signals were normalized to that of Ponceau S staining. Expression in control cells after 3 days of differentiation was set as 1. Data represent the mean ± SD of three independent experiments. Asterisk indicates statistical significance compared to the control sample. The membranes in (**A**) and (**C**) were cut prior to hybridization with antibodies. Full-length blots are presented in Supplementary Fig. 9.

We further examined the effect of ZSWIM8 overexpression on C2C12 differentiation. 3 × FLAG-ZSWIM8-expressing C2C12 cells were differentiated for up to 3 days (Fig. 5A and B). As expected, ZSWIM8 overexpression prevented C2C12 differentiation, which was indicated by weak induction of MHC and myogenin. Unexpectedly, the expression and induction of Cdon were not affected by ZSWIM8 overexpression. ZSWIM8 is a ubiquitin ligase that interacts with Cdon, but it might not target Cdon for proteasomal degradation, or endogenous ZSWIM8 might be enough to downregulate Cdon.

Subsequently, we examined Cdon-dependent C2C12 differentiation using two independent Cdon-knockdown cell lines by utilizing two different target sequences, *Cdon*#18 and *Cdon*#19 (Fig. 5C and D). These cell lines were differentiated for up to 3 days, and were monitored based on MHC and myogenin expression. As reported previously^16–18^, Cdon knockdown prevented C2C12 differentiation, as shown by the decreased MHC expression. The expression of Bnip-2 and JLP was not affected by Cdon knockdown. Notably, endogenous Cdon was almost completely knocked down; however, its expression was partly recovered during C2C12 differentiation. Altogether, we propose that ZSWIM8 prevents C2C12 differentiation without affecting Cdon expression.

### Cleavage of Cdon by proteasome inhibitor MG132

**Figure 6:**
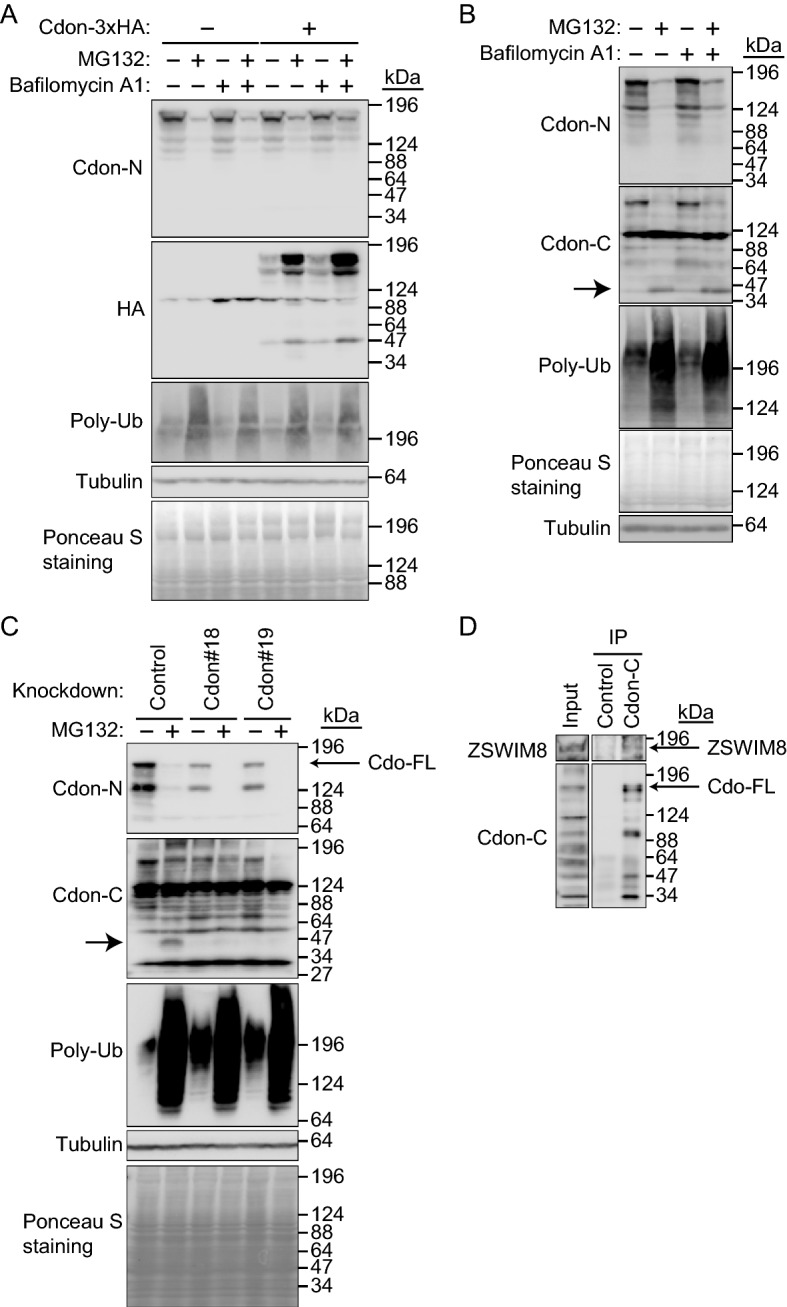
Downregulation of full-length Cdon by the proteasome inhibitor MG132 but not by the lysosome inhibitor bafilomycin A1. (**A**) Downregulation and accumulation of endogenous Cdon and Cdon-3HA, respectively, with the proteasome inhibitor MG132. Cdon-3 × HA was transiently expressed in C2C12 cells and cultured with or without MG132 (10 μM) and/or bafilomycin A1 (0.5 μM) for 7 h. The cell lysates were subjected to immunoblotting with an anti-Cdon-N, HA, or polyubiquitin (Poly-Ub) antibody. Tubulin and Ponceau S staining were used as loading controls. Representative data of three independent experiments. (**B**) Accumulation of Cdon fragment with MG132. C2C12 cells were cultured with or without MG132 (10 μM) and/or bafilomycin A1 (0.5 μM) for 7 h. The cell lysates were subjected to immunoblotting with an anti-Cdon-N, Cdon-C, or Poly-Ub antibody. Tubulin and Ponceau S staining were used as loading controls. The MG132-dependent Cdon fragment is indicated by an arrow. Representative data of three independent experiments. (**C**) Downregulation of Cdon fragment by Cdon knockdown. Control or Cdon-knockdown (#18 or 19) C2C12 cells were cultured with or without MG132 (10 μM) for 7 h. The cell lysates were subjected to immunoblotting with an anti-Cdon-N, Cdon-C, Poly-Ub, or Tubulin antibody. Tubulin and Ponceau S staining were used as loading controls. The MG132-dependent Cdon fragment is indicated by an arrow. Representative data of three independent experiments. (**D**) Endogenous interaction between ZSWIM8 and Cdon. C2C12 cells were differentiated for 4 days. The cell lysates were subjected to immunoprecipitation with an anti-Cdon-C or control antibody and immunoblotted with an anti-ZSWIM8 or Cdon-C antibody. Representative data of two independent experiments. The membranes in (**A**) to (**D**) were cut prior to hybridization with antibodies. Full-length blots are presented in Supplementary Fig. 10.

We next examined the stability of Cdon. C-terminal 3 × HA-tagged Cdon (Cdon-3 × HA) was transiently expressed in C2C12 cells, which were treated with the potent proteasome inhibitor MG132 or lysosome inhibitor bafilomycin A1 (Fig. 6A). If Cdon was degraded by a proteasome or lysosome, it would be accumulated with one of these inhibitors. Unexpectedly, MG132 treatment downregulated endogenous Cdon expression, whereas bafilomycin A1 did not. In contrast, exogenous Cdon-3 × HA was accumulated with MG132 treatment but not with bafilomycin A1. Since the transfection efficiency was relatively low, Cdon-3 × HA overexpression did not apparently increase total Cdon expression, as detected by the anti-Cdon-N antibody. These data suggested that the C-terminal tagging of Cdon affected the overall degradation pathway. Nevertheless, we examined the possible ubiquitination of Cdon-3 × HA by ZSWIM8 with His_6_-ubiquitin (His_6_-Ub) (Supplementary Fig. 4). 3 × FLAG-ZSWIM8 and Cdon-3 × HA were expressed in HEK293T cells, which were then treated with MG132 and lysed with 8 M urea-containing lysis buffer to dissociate all protein–protein interactions. His_6_-Ub and covalently His_6_-Ub-modified proteins were pulled down using Ni-agarose and subjected to SDS-PAGE and IB with an anti-HA, anti-FLAG, or anti-His_6_ antibody. Results showed that Cdon-3 × HA was polyubiquitinated. However, it was not increased with the co-expression of 3 × FLAG-ZSWIM8, indicating that ZSWIM8 would not target Cdon-3 × HA.

We speculated that MG132 might break down Cdon by an unknown mechanism because the anti-Cdon-N antibody, which we used, recognizes the external domain of Cdon. If the transmembrane regions were cleaved and the external domain of Cdon was released from the cell membrane, we would not be able to detect Cdon using the anti-Cdon-N antibody. In this case, its cytoplasmic domain (CD) would still exist in the cell, which might be recognized by ZSWIM8 for ubiquitination and proteasomal degradation. Therefore, we used an anti-Cdon-C antibody that recognizes the CD. C2C12 cells were treated with MG132 or bafilomycin A1, and Cdon was detected using an anti-Cdon-N or anti-Cdon-C antibody (Fig. 6B). As expected, IB with an anti-Cdon-C antibody showed accumulation of the Cdon fragment at approximately 40 kDa after MG132 treatment. This indicates that MG132 treatment indirectly cleaves Cdon, and the resulting CD is degraded by the proteasome. To confirm that the ~ 40 kDa band observed in the blot is a Cdon fragment, control and two Cdon-knockdown cell lines were treated with MG132 and subjected to IB with anti-Cdon-C antibody (Fig. 6C). The ~ 40 kDa band was downregulated by Cdon knockdown, suggesting that it was a Cdon fragment. We next examined the interaction between endogenous ZSWIM8 and Cdon by utilizing the Cdon-C antibody (Fig. 6D). C2C12 cells were differentiated for 4 days and subjected to IP-Western blotting with anti-ZSWIM8 and anti-Cdon-C antibodies. Our results indicated that anti-Cdon-C antibody immunoprecipitated endogenous ZSWIM8. Therefore, we examined ZSWIM8-dependent polyubiquitination of CD with His_6_-Ub in a HEK293T overexpression system (Supplementary Fig. 5). 3 × FLAG-ZSWIM8 and Cdon(FL or CD) with no-tag were expressed in HEK293T cells, which were then treated with MG132 and lysed with 8 M urea-containing lysis buffer. His_6_-Ub and covalently His_6_-Ub-modified proteins were pulled down using Ni-agarose and subjected to SDS-PAGE and IB with an anti-Cdon-C, anti-FLAG, or anti-His_6_ antibody. First, full-length Cdon with no-tagging was not cleaved by MG132 in HEK293T cells. Therefore, cleavage of Cdon by MG132 (Fig. 6) could be cell type-specific. Our results also showed that Cdon(CD) was polyubiquitinated, but the ubiquitination was not increased by the co-expression of 3 × FLAG-ZSWIM8, indicating that ZSWIM8 would not target Cdon(CD). Altogether, if Cdon(CD) was produced, a proteasome would degrade it independently of ZSWIM8.

### ZSWIM8 is included in the Cdon complex

**Figure 7:**
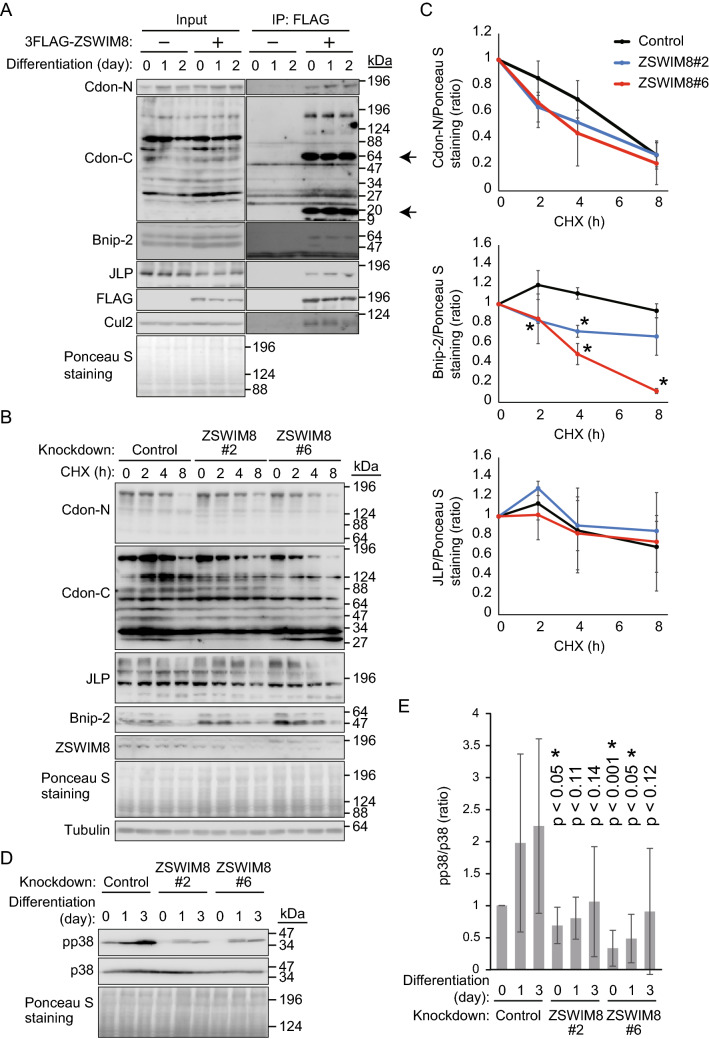
No effect on p38α/β MAPK by ZSWIM8 knockdown during C2C12 differentiation. (**A**) Control or 3 × FLAG-ZSWIM8-expressing C2C12 cells were differentiated for 1 or 2 days. The cell lysates were subjected to immunoprecipitation with an anti-FLAG antibody and immunoblotting with an anti-Cdon-N, Cdon-C, Bnip-2, JLP, FLAG, or Cul2 antibody. Cul2 and Ponceau S staining were used as loading controls. ZSWIM8-interacting Cdon fragments are indicated by arrows. Representative data of two independent experiments. (**B**) ZSWIM8 does not affect protein stabilities of Cdon, JLP, and Bnip-2. Control or ZSWIM8-knockdown C2C12 cells were differentiated for 3 days. The cells were further cultured with or without cycloheximide (CHX, 50 μg/mL) for the indicated time (h). The cell lysates were subjected to immunoblotting with an anti-Cdon-N, Cdon-C, JLP, Bnip-2, or ZSWIM8 antibody. Tubulin and Ponceau S staining were used as loading controls. Representative data of three independent experiments are shown. (**C**) Quantification of Cdon, Bnip-2, and JLP in (**B**). Cdon, Bnip-2, or JLP signals were normalized to that of Ponceau S staining. Expression in time = 0 was set as 1. Data represent the mean ± SD of three independent experiments. (**D**) Possible downregulation of p38α/β MAPK phosphorylation by ZSWIM8 knockdown during C2C12 differentiation. Control or ZSWIM8-knockdown C2C12 cells were differentiated for 1 or 3 days. The cell lysates were subjected to immunoblotting with an anti-phospho-p38α/β MAPK (pp38) or total p38α/β MAPK antibody (p38). p38 and Ponceau S staining were used as loading controls. Representative data of five independent experiments are shown. (**E**) Quantification of p38α/β MAPK phosphorylation in (**D**). pp38 signals were normalized to those of p38. The phosphorylation ratio in control cells without differentiation was set as 1. Data represent the mean ± SD of five independent experiments. Asterisk indicates statistical significance compared to the control sample. The membranes in (**A**), (**B**), and (**D**) were cut prior to hybridization with antibodies. Full-length blots are presented in Supplementary Fig. 11.

Next, we examined the interaction between ZSWIM8 and members of the Cdon complex, namely Bnip-2 and JLP. 3 × FLAG-ZSWIM8-expressing C2C12 cells were cultured with or without differentiation for up to 2 days. The cell lysates were then subjected to IP with an anti-FLAG antibody, and the immunoprecipitates were analyzed with an anti-Cdon-C, Cdon-N, Bnip-2, JLP, or anti-FLAG antibody (Fig. 7A). Apart from Cdon, ZSWIM8 interacted with Bnip-2 and JLP. The anti-Cdon-C antibody revealed strong interactions between ZSWIM8 and a ~ 64 kDa protein and a ~ 20 kDa protein, possibly Cdon cytoplasmic fragments, in addition to interactions with Cdon(FL). ZSWIM8 also interacted with Cul2 as expected and is suggested to be a Cul2-type ubiquitin ligase and might target Bnip-2 and/or JLP for ubiquitination and proteasomal degradation instead of Cdon. In this case, ZSWIM8 knockdown should stabilize these proteins. To examine this hypothesis, control and ZSWIM8-knockdown C2C12 cells were differentiated for 3 days, and protein translation was inhibited by cycloheximide to analyze the stability of Cdon, JLP, and Bnip-2 (Fig. 7B and C). However, ZSWIM8 knockdown did not affect the stability of these proteins, indicating that ZSWIM8 is not a ubiquitin ligase for Cdon, JLP, and Bnip-2. Given that the Cdon/JLP/Bnip-2 complex activates p38α/β MAPK and induces differentiation^20^, we examined the activity of p38α/β MAPK by analyzing its phosphorylation status. Control and ZSWIM8-knockdown C2C12 cells were differentiated for up to 3 days, and cell lysates were analyzed with an anti-phospho or total p38α/β MAPK antibody (Fig. 7D). p38α/β MAPK was phosphorylated with differentiation in a time-dependent manner. Importantly, ZSWIM8 knockdown tended to downregulate the phosphorylation of p38α/β MAPK. These experiments were repeated five times and statistically compared with the results using control cells (Fig. 7E). Nevertheless, the error bars were relatively large, and no statistical significance, especially on day 3, was observed. These data suggest that this downregulation of p38α/β MAPK phosphorylation is not regulated directly by ZSWIM8, rather, several other pathways may be involved. Altogether, ZSWIM8 is included in the Cdon complex but does not regulate p38α/β MAPK activation during C2C12 differentiation under the conditions examined. Another possibility is that the phosphorylation of p38α/β MAPK was indirectly downregulated by accelerated C2C12 differentiation via an unidentified pathway, which was achieved by ZSWIM8 knockdown.

## Discussion

The balance between self-renewal and differentiation of satellite cells is strictly regulated to maintain the satellite cell pool. p38α MAPK restricts the excess proliferation of satellite cells in the postnatal growth phase while promoting timely myoblast differentiation^21^. Muscle regeneration after cardiotoxin-induced injury is delayed in the absence of p38α MAPK^21^, and thus, p38α MAPK is recognized as a master kinase during the differentiation of satellite cells^2^. When satellite cells are activated, for example, by muscle injury, they enter the cell cycle where a subset undergoes asymmetric division. p38α/β MAPK is phosphorylated and activated in only one daughter cell, in which the transcription factor myogenic differentiation 1 is induced. This daughter cell enters the cell cycle and generates a proliferating myoblast. In contrast, the other daughter cell renews the quiescent satellite cell pool without active p38α/β MAPK^22^. Therefore, p38α/β MAPK activation is dynamically regulated when satellite cells differentiate into myoblasts. Here, we propose that ZSWIM8 is included in the Cdon complex. However, there is no correlation between ZSWIM8 expression and p38α/β MAPK activation during C2C12 myoblast differentiation (Fig. 7D and E).

ZSWIM8 contains a BC box and Cul2 box, and is thus recognized as a ubiquitin ligase^10,13^. Although ZSWIM8 interacts with Cdon, Bnip-2, and JLP, its knockdown did not affect the stability of these proteins. Therefore, it may regulate signal transduction through the Cdon complex via binding but not via ubiquitin ligase activity. The family of suppressor of cytokine signaling (SOCS) proteins contains a SOCS box, which interacts with Cul5, Elongin B, and Elongin C, and forms a protein complex with ubiquitin ligase activity. SOCS proteins ubiquitinate signaling molecules, including cytokine receptors and Janus kinase, and lead to their proteasomal degradation. In contrast, SOCS proteins bind to phosphotyrosine residues on cytokine receptors via their Src homology 2 domain, thereby competing with signal transducer and activator of transcription binding and activation. ZSWIM8 might also block signal transduction similar to the SOCS protein. ZSWIM8 interacts with Argonaute proteins and regulates microRNAs (miRNAs) expression in multiple cell types in collaboration with Cul3^23,24^. This finding suggests two independent functions of ZSWIM8, such as the myogenic function in collaboration with Cul2 and the regulation of miRNAs with Cul3.

Full-length Cdon is degraded in the presence of the proteasome inhibitor MG132 (Fig. 6). Cdon could be cleaved inside the cell membrane due to the anti-Cdon-N antibody that recognizes the external domain of Cdon but does not recognize Cdon after MG132 treatment. Meanwhile, the anti-Cdon-C antibody, which recognizes the cytoplasmic region of Cdon, also recognizes Cdon after MG132 treatment. The cleavage of transmembrane proteins inside the cell membrane is a well-known mechanism for signal transduction. For example, ligand binding to the Notch transmembrane receptor induces the cleavage of Notch by proteases of a disintegrin and metalloprotease domain family, as well as the presenilin-dependent gamma-secretase complex inside the cell membrane. Thereafter, the Notch intracellular domain is released into the cytoplasm and then translocated to the nucleus to regulate the expression of several target genes^25,26^. Although whether Cdon is cleaved by the same proteases is not clear; its cytoplasmic region is cleaved by caspase-3 at Asp1153, and the resulting Cdon (1–1153), including the external transmembrane and a part of the cytoplasmic region, induces apoptosis^27^.

Cdon binds Sonic hedgehog (Shh) and is involved in the hedgehog signaling pathway^28–30^. Cdon also interacts with the cell–cell adhesion molecule N-cadherin^31^. N-cadherin ligation activates p38α/β MAPK in a Cdon-, JLP-, and Bnip-2-dependent manner^32^. On the contrary, neither JLP nor Bnip-2 is associated with Cdon bound to Shh, and Shh does not activate p38α/β MAPK in myoblasts^32^, suggesting that N-cadherin ligation is a major initiator of myoblast differentiation.

Both Cdon and brother of Cdon (Boc) are cell surface glycoproteins of the immunoglobulin (Ig)/fibronectin type III-like (FNIII) family and have similar ectodomain structures^33,34^. Interactions between the Boc ectodomain, but not the intracellular domain, and Cdon positively regulate myoblast differentiation^33^. However, whether ZSWIM8 associates with these initiators, such as Shh, N-cadherin, and Boc, to regulate myoblast differentiation is unclear.

Altogether, ZSWIM8 is induced during C2C12 differentiation and may be included in the Cdon complex, composed of Cdon, Bnip-2, and JLP (Supplementary Fig. 6). ZSWIM8 neither affects the stability of these proteins nor regulates p38α/β MAPK activation, while its knockdown may downregulate p38α/β MAPK and enhance C2C12 differentiation. It would be possible that several pathways are involved in this regulation, while the resulting enhanced C2C12 differentiation may downregulate p38α/β MAPK phosphorylation indirectly.

Whether production of the Cdon cytoplasmic fragment is dependent on ligand activation is unclear since proteasome inhibition is required for the detection of the Cdon cytoplasmic fragment, and proteasome inhibition artificially led to the production of the cytoplasmic fragment. The cytoplasmic fragment might form a complex including that with ZSWIM8 to maintain the phosphorylation status of p38α/β MAPK. However, examining the unstable Cdon cytoplasmic fragment-containing complex is challenging and remains to be investigated.

## Limitations of the study

We did not detect the ZSWIM8-dependent ubiquitination of Cdon. However, this does not indicate that ZSWIM8 is not a ubiquitin ligase targeting Cdon; rather, this might have been due to the low levels of Cdon being ubiquitinated in C2C12 cell.; However, ZSWIM8 might target Cdon for proteasomal degradation in different tissues. Furthermore, the anti-Cdon-C polyclonal antibody showed high background, which made it difficult to examine cellular localization. Alternatively, C-terminal tagging could affect the conformation of Cdon. Thus, a better selective antibody that recognizes the Cdon cytoplasmic fragment should be produced for further analysis to elucidate the ZSWIM8-dependent signaling pathway.

## Materials and methods

### Reagents

Protein A Sepharose was purchased from GE Healthcare Bio-Sciences. MG132 was purchased from Calbiochem. Cycloheximide was purchased from Nacalai Tesque. Ethylenediaminetetraacetic acid (EDTA), cOmplete EDTA-free protease inhibitor cocktail, 4′,6-diamidino-2-phenylindole (DAPI), polybrene, Ponceau S, Triton X-100, and Tween-20 were purchased from Merck. Can Get Signal was purchased from Toyobo. Bafilomycin A1, Ni–NTA agarose, and gelatin were purchased from Fujifilm Wako Pure Chemical Corporation.

### Plasmid construction

Human *Zswim8* (GenBank/EBI accession number: NM_015037) and mouse *Cdon* (NM_021339) cDNA were PCR-amplified from a HeLa and C2C12 cell cDNA library, respectively, and subcloned into pcDNA3-puro containing a 3 × FLAG sequence or pCI-neo containing a 3 × HA sequence. pMT107 expresses N-terminal hexa-histidine (His_6_)-tagged-ubiquitin^35^.

### Gene knockdown with short hairpin RNA (shRNA)

The pMX-puro II vector was constructed by deleting the U3 portion of the 3′-long terminal repeat of pMX-puro. The mouse U6 gene promoter followed by DNA corresponding to an shRNA sequence was subcloned into the NotI and XhoI sites of pMX-puro II, yielding pMX-puro II-U6/siRNA. The DNA for the shRNA encoded a 21-nucleotide hairpin sequence specific to the target mRNA, with a loop sequence (-TTCAAGAGA-) separating the two complementary domains and contained a tract of five T nucleotides to terminate transcription ^12^. Double-stranded oligo DNA for the shRNA was ligated into the pMX-puro II vector. Mouse *Zswim8* #2 and #6 target sequences were 5′-GAGCTTCCTCAGCAGATTCTC-3′ and 5′-GAAGTCTAGGGTTGGGGGAGA-3′, respectively, and those for mouse *Cdon* #18 and #19 were 5′-GAAACAAAGTTGTCTCAGAGT-3′ and 5′-GAGTTTGAGTTCTCCTTATTG-3′, respectively. The non-specific target sequence was 5′-CAGTCGCGTTTGCGACTGG-3′^36^. Retroviruses encoding the shRNAs were produced by transfecting pMX-puro II and pCL-10A1 into HEK293T cells. Two days after transfection, a culture medium containing recombinant retroviruses was collected^37^. C2C12 cells were cultured in the culture medium containing recombinant retroviruses in the presence of polybrene (10 μg/mL) for 2 days, and then selected with puromycin (3 μg/mL) for 1 week.

### Cell culture and transduction

HEK293T, 786-O, K562, RCC4, and U-2 OS cells (American Type Culture Collection, ATCC) were cultured in DMEM (Fujifilm Wako Pure Chemical Corporation) supplemented with 10% fetal bovine serum (FBS), penicillin G (100 units/mL), and streptomycin (100 μg/mL). C2C12 cells were a gift from Dr. Masafumi Kuzuya (Nagoya University, Japan). C2C12 cells (RCB0987, lot #41) were provided by the RIKEN BRC through the National BioResource Project of the MEXT/AMED, Japan. MCF10A cells were cultured in DMEM/Ham’s F-12 (Fujifilm Wako Pure Chemical Corporation) supplemented with 5% FBS, 100 units/mL penicillin G (Nakalai, Japan), 100 μg/mL streptomycin (Nakalai, Japan), 20 ng/mL epidermal growth factor (Novus Biologicals, USA), 10 μg/mL insulin (Roche, Germany), 1 ng/mL cholera toxin (Bio Academia, Japan), and 1 μg/mL hydrocortisone (Tokyo Chemical Industry). Confluent C2C12 cells on gelatin-coated dishes were differentiated into myotubes by replacing 10% fetal calf serum (FCS) with 2% horse serum. The medium was changed every day for the indicated periods. MG132 (2 μM) was added to the culture medium for the indicated period. HEK293T and C2C12 cells were transfected with expression plasmids using polyethyleneimine (MW-25 K; Polysciences Inc., USA) at a mass ratio of 1:3.

### Antibodies

A rabbit polyclonal antibody to ZSWIM8 was generated by immunizing rabbits with the peptide CSSQRGPRRLSAEGGDKALH, corresponding to amino acids 558–576 of human ZSWIM8, by Merck (Japan). The anti-ZSWIM8 antibody was purified from antisera by a glutathione S-transferase (GST)-tagged peptide and used for IB (2.5 μg/mL in PBS). A rabbit polyclonal antibody to the C-terminus of mouse Cdon (Cdon-C) was generated by immunizing rabbits with the peptide HLVNSGGVYTAVPQMDPLEC, corresponding to amino acids 1080–1099 of mouse Cdon, by Merck (Japan). The anti-Cdon-C antibody was purified from antisera by GST-Cdo(CD) and used for IB (5 μg/mL in Can Get Signal). A rabbit polyclonal antibody to HA was generated in our laboratory and used for IB (1 μg/mL in PBS). Antibodies against the following proteins were also used: FLAG (1 μg/mL in PBS; M2; Sigma-Aldrich), MHC (sarcomere; 1:100 dilution in Can Get Signal; MF 20; Developmental Studies Hybridoma Bank), myogenin (1:100 dilution in Can Get Signal; F5D; Developmental Studies Hybridoma Bank), Cdon-N (0.2 μg/mL in Can Get Signal; AF2429; R&D systems), Bnip-2 (1 μg/mL in PBS; GTX30091; GeneTex), JLP (1 μg/mL in Can Get Signal; ab12331; Abcam), poly-ubiquitin (1 μg/mL in Can Get Signal; FK2; Cosmo Bio), Cul2 (1 μg/mL in PBS; sc-166506; Santa Cruz Biotechnology), total and phosphorylated p38 MAPK (1 μg/mL in PBS; PhosphoPlus p38 MAPK (Thr180/Tyr182) Antibody Duet #8203; Cell Signaling), S6 ribosomal protein (1 μg/mL in PBS; 5G10; Cell Signaling), β-tubulin (0.3 μg/mL in PBS; E1C601; EnoGene), Mitofusin 2 (1 μg/mL in Can Get Signal; M6444; Merck) and His_6_ (1 μg/mL in PBS; MAB050; R&D systems).

### IP and IB analyses

Cells were lysed in lysis buffer containing 50 mM Tris–HCl (pH 7.4), 150 mM NaCl, 1% Triton X-100, 0.4 mM Na_3_VO_4_, 0.4 mM EDTA, 10 mM NaF, 10 mM sodium pyrophosphate, and Complete EDTA-free protease inhibitor cocktail (according to the manufacturer’s protocol). The lysates were incubated with 0.5 μg of antibody overnight and then with 10 μL of Protein A Sepharose beads at 4 °C for 1 h. The beads were washed three times with lysis buffer. Immunoprecipitated proteins were eluted using the SDS-PAGE sample buffer with boiling, separated by SDS-PAGE, and transferred onto a nitrocellulose membrane (Protran NC 0.1; 10,600,000; Cytiva). The membrane was incubated in 3% skim milk in PBS at room temperature for 15 min and then with primary antibodies at 4 °C overnight. The membrane was washed three times with Tris-buffered saline with Tween-20 (TBST) containing 50 mM Tris–HCl (pH 7.4), 150 mM NaCl, and 0.1% Tween-20 at room temperature for 15 min, followed by incubation with horseradish peroxidase-conjugated anti-goat IgG (1:4000 dilution in 3% skim milk in PBS; sc-2020; Santa Cruz Biotechnology), anti-mouse IgG (1:8000 dilution in 3% skim milk in PBS; A4416; Merck), or anti-rabbit IgG (1:8000 dilution in 3% skim milk in PBS; A6154; Merck) at room temperature for 1 h. The membrane was washed three times with TBST for 15 min. Protein levels were measured using the enhanced chemiluminescence reagent (Luminata Forte, Merck or Chemi-Lumi One L, Nacalai Tesque) and the SOLO.7S.EDGE (Vilber, France) system. Images were processed using Photoshop but were not cropped from different parts of the same gel, or from different gels or fields.

### Ni-agarose pull-down

HEK293T cells cultured with or without MG132 (2 μM for 15 h) in 6 cm dishes were sonicated (UD-211; Tomys Seiko) in PBS containing 8 M urea and 10 mM imidazole. After centrifugation, the supernatant was incubated with 10 μL of Ni–NTA agarose beads at 4 °C for 1 h in the presence of 1% Triton X-100. The beads were washed three times with the aforementioned buffer containing 1% Triton X-100. His_6_-ubiquitinated proteins were eluted using the SDS-PAGE sample buffer containing 300 mM imidazole with boiling.

### Immunofluorescence staining

C2C12 cells were fixed in 4% paraformaldehyde in PBS at room temperature for 15 min and washed three times with PBS. The cells were incubated with antibodies against MHC, FLAG (both 1 μg/mL in PBS), or Cdon-N (1 μg/mL in Can Get Signal), in the presence of 0.1% Triton X-100 and 0.1% bovine serum albumin at 4 °C overnight, and then washed three times with PBS. The cells were incubated with Alexa Fluor 594 goat anti-rabbit and Alexa Fluor 488 goat anti-mouse antibodies (both at a 1:2000 dilution; Invitrogen) in PBS containing 0.1% Triton X-100 and 0.1% bovine serum albumin in the dark at room temperature for 1 h. Then, the cells were incubated with 0.1 μg/mL DAPI in PBS for 1 min, washed extensively with PBS, and photographed using a charge-coupled device camera (Axio Observer Z1; Zeiss) or a confocal microscope (Eclipse Ti-C2; Nikon).

### Statistical analysis

Data are reported as the mean ± standard deviation (SD) of three or five independent experiments. Means of different groups were compared using Student’s *t*-test. *P *value < 0.05 was considered statistically significant.

### Human and animals rights

Animals were not used in the current study.

## Supplementary Information

Supplementary Information 1.

Supplementary Information 2.

Supplementary Information 3.

Supplementary Information 4.

Supplementary Information 5.

Supplementary Information 6.

Supplementary Information 7.

Supplementary Information 8.

Supplementary Information 9.

Supplementary Information 10.

Supplementary Information 11.

Supplementary Information 12.

Supplementary Information 13.

Supplementary Information 14.

Supplementary Information 15.

## Data Availability

No datasets were generated during the current study.
