# Supplementary material for: ZSWIM8 is a myogenic protein that partly prevents C2C12 differentiation
Source: Sci Rep. 2021 Oct 22;11:20880. doi: 10.1038/s41598-021-00306-6 (PMC8536758; doi:10.1038/s41598-021-00306-6)
Supplement: Supplementary file 1 — Supplementary Information 1. [file 41598_2021_306_MOESM1_ESM.pdf]

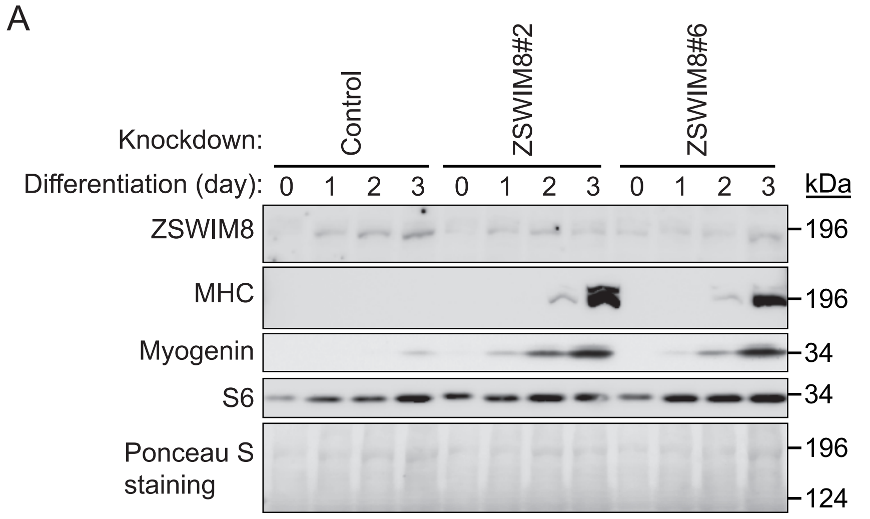

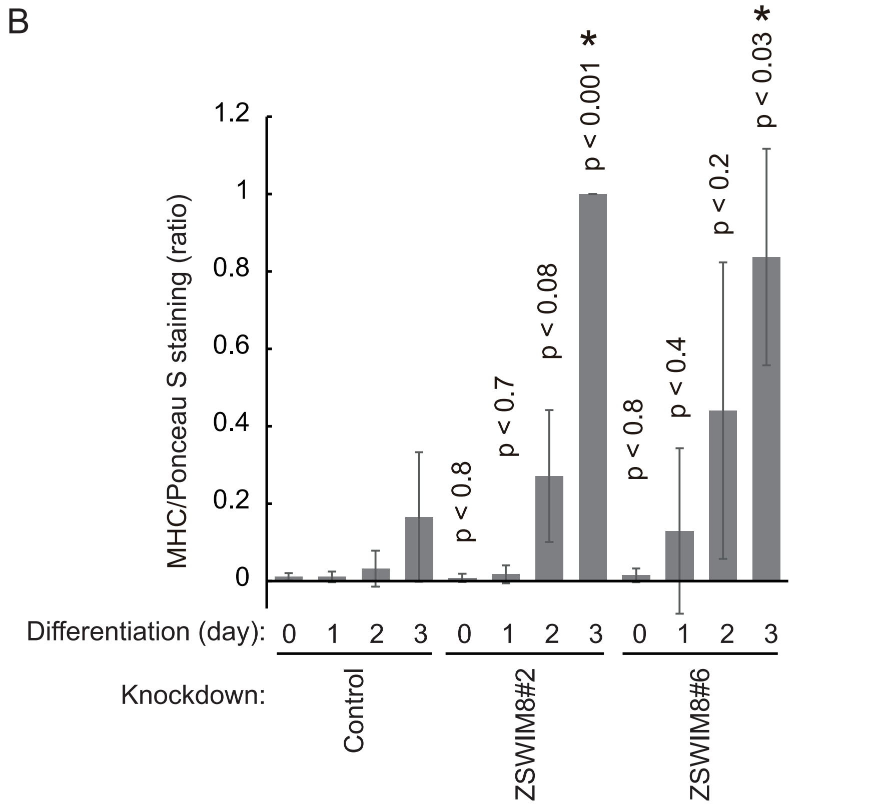

Supplementary Figure 1. Prevention of C2C12 differentiation by ZSWIM8

(A) Prevention of C2C12 differentiation by ZSWIM8. Control or ZSWIM8-knockdown (#2 and #6) C2C12 cells were differentiated for 1, 2, or 3 days. The cell lysates were subjected to immunoblotting with an anti-ZSWIM8, myosin heavy chain (MHC), or myogenin antibody. S6 ribosomal protein and Ponceau S staining were used as loading controls. Representative data of three independent experiments. The membranes were cut prior to hybridization with antibodies. The original blots are shown in the "Supplementary information" file. (B) Quantification of MHC expression in (A). MHC signals were normalized to that of Ponceau S staining. Expression in ZSWIM8-knockdown#2 cells after 3 days of differentiation was set as 1. Data represent the mean ± SD of three independent experiments. Asterisk indicates statistical significance compared to the control sample. The membranes in (A) were cut prior to hybridization with antibodies. Full-length blots are presented in Supplementary Figure 11.
