# Supplementary material for: ZSWIM8 is a myogenic protein that partly prevents C2C12 differentiation
Source: Sci Rep. 2021 Oct 22;11:20880. doi: 10.1038/s41598-021-00306-6 (PMC8536758; doi:10.1038/s41598-021-00306-6)
Supplement: Supplementary file 2 — Supplementary Information 2. [file 41598_2021_306_MOESM2_ESM.pdf]

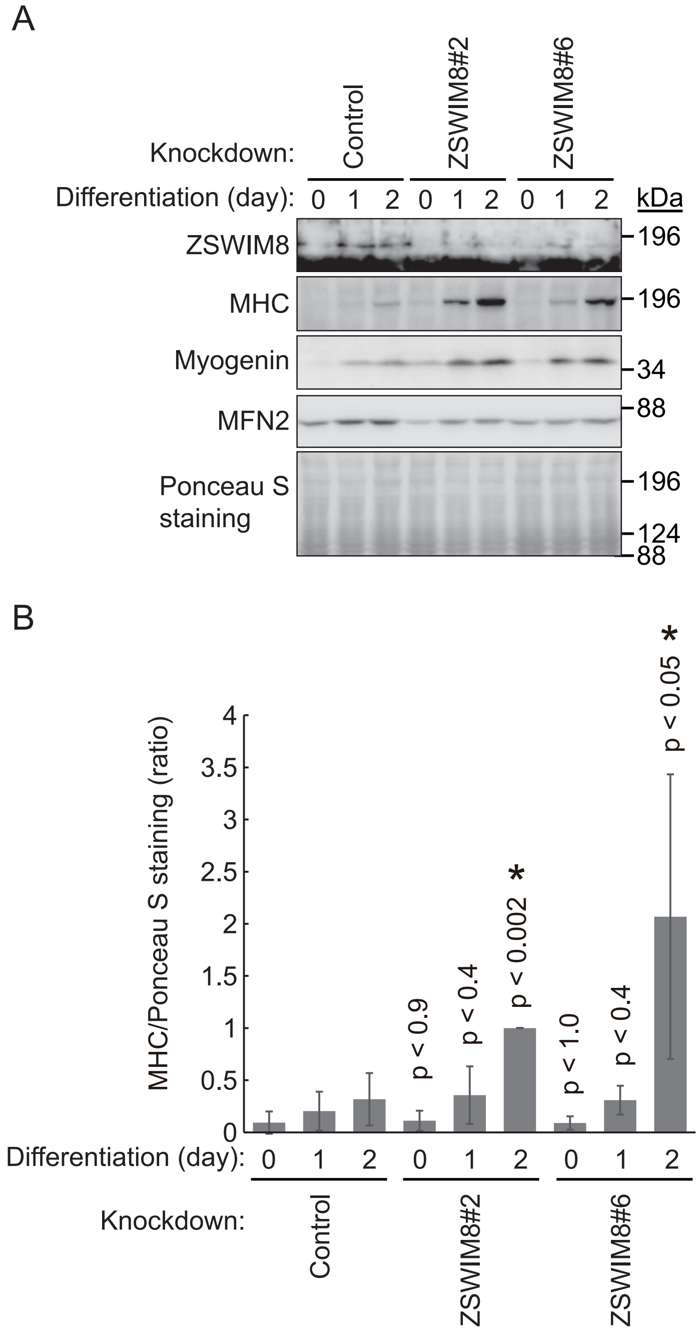

Supplementary Figure 2. Prevention of C2C12 differentiation by ZSWIM8

(A) Prevention of C2C12 differentiation by ZSWIM8. Control or ZSWIM8-knockdown (#2 and #6) C2C12 cells were differentiated for 1 or 2 days. The cell lysates were subjected to immunoblotting with an anti-ZSWIM8, myosin heavy chain (MHC), or myogenin antibody. Mitofusin 2 (MFN2) and Ponceau S staining were used as loading controls. Representative data of four independent experiments. The membranes were cut prior to hybridization with antibodies. Full-length blots are presented in Supplementary Figure 13. (B) Quantification of MHC expression in (A). MHC signals were normalized to that of Ponceau S staining. Expression in ZSWIM8-knockdown#2 cells after 2 days of differentiation was set as 1. Data represent the mean ± SD of four independent experiments. Asterisk indicates statistical significance compared to the control sample.
