# Supplementary material for: ZSWIM8 is a myogenic protein that partly prevents C2C12 differentiation
Source: Sci Rep. 2021 Oct 22;11:20880. doi: 10.1038/s41598-021-00306-6 (PMC8536758; doi:10.1038/s41598-021-00306-6)
Supplement: Supplementary file 3 — Supplementary Information 3. [file 41598_2021_306_MOESM3_ESM.pdf]

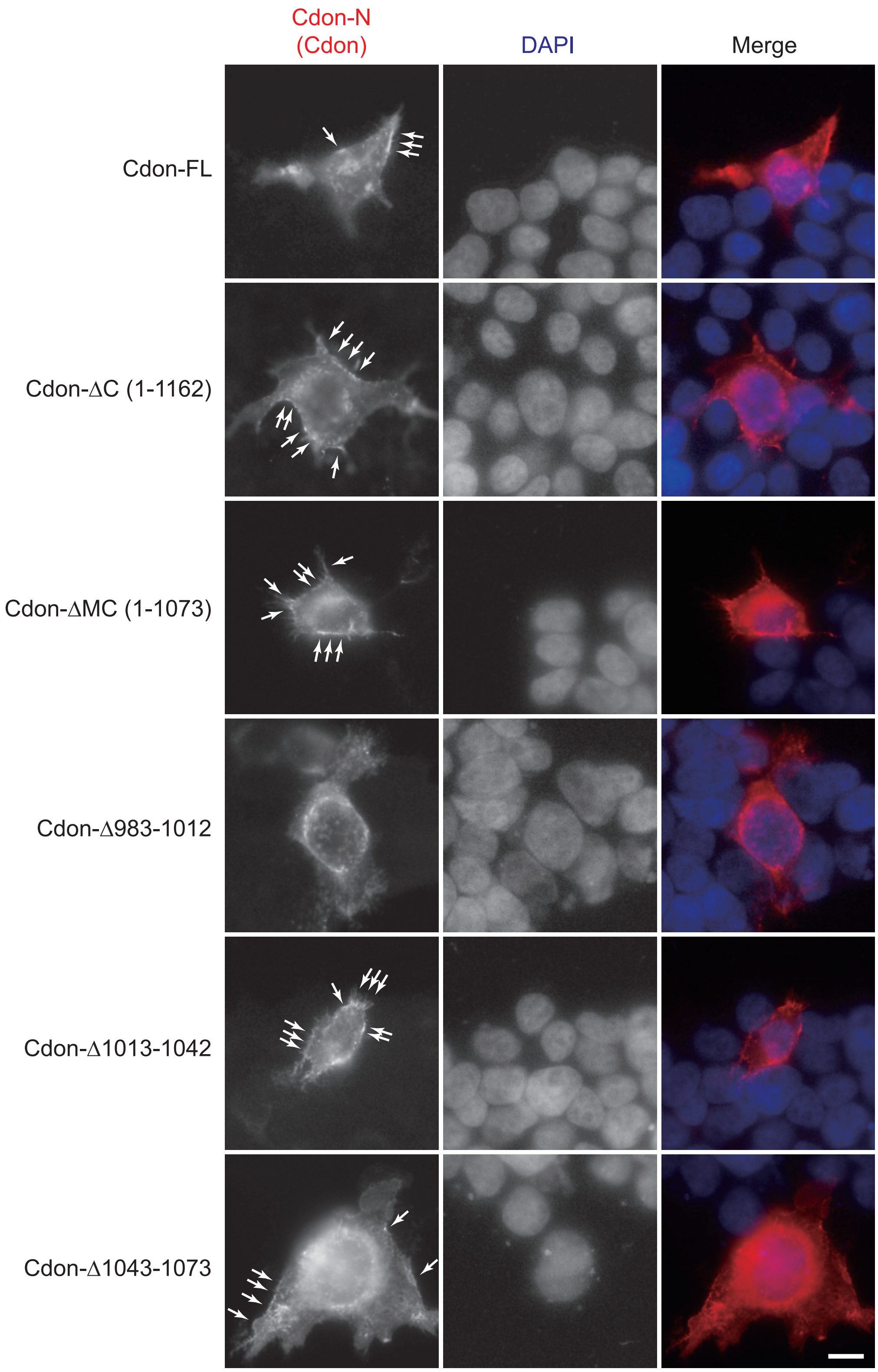

Supplementary Figure 3. Cellular localization of Cdon (full length and deletion mutants)

Transfected HEK293T cells were immunostained with an anti-Cdon-N antibody. Nuclei were stained with DAPI. Scale bar, 10 μm. Representative localization to the cell membrane is indicated by the white arrow.
