# Supplementary material for: ZSWIM8 is a myogenic protein that partly prevents C2C12 differentiation
Source: Sci Rep. 2021 Oct 22;11:20880. doi: 10.1038/s41598-021-00306-6 (PMC8536758; doi:10.1038/s41598-021-00306-6)
Supplement: Supplementary file 4 — Supplementary Information 4. [file 41598_2021_306_MOESM4_ESM.pdf]

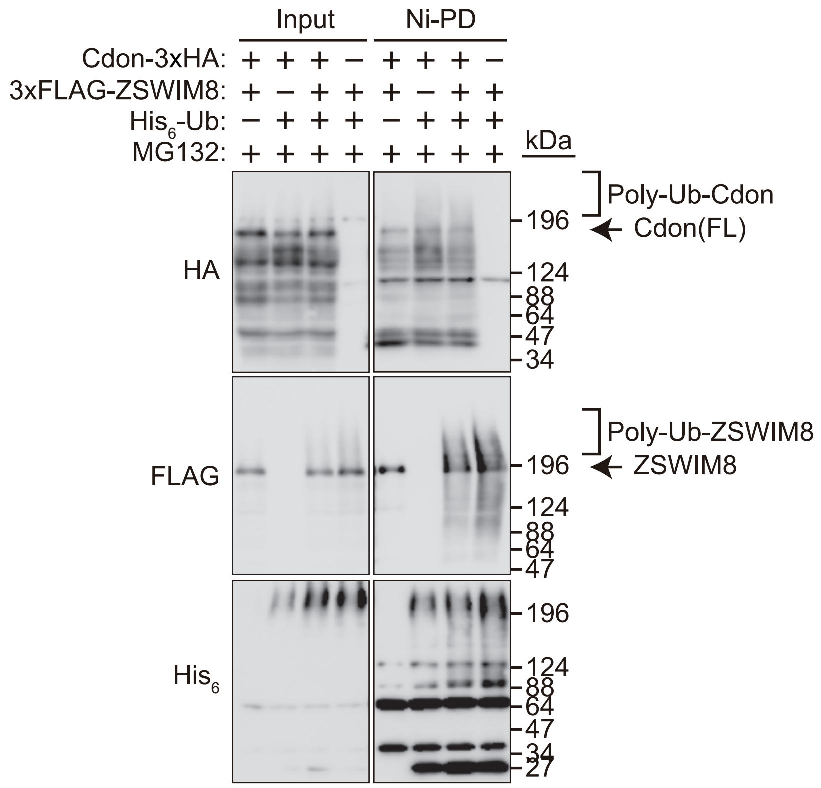

Supplementary Figure 4. Overexpression of ZSWIM8 does not induce ubiquitination of Cdon

His_6-ubiquitination of Cdon is not dependent on the ZSWIM8 overexpression. Cdon-3×HA, 3×FLAG-ZSWIM8, and His_6-ubiquitin (Ub) were expressed in HEK293T cells with the indicated combinations. The cells were cultured in the presence of MG132 (2 μM for 15 h) before harvest. The cell lysates containing 8 M urea, which prevents protein–protein interactions, were subjected to Ni-NTA agarose pull down (Ni-PD) to purify proteins modified by His6-Ub, followed by immunoblot analysis with an anti-HA, anti-FLAG, or anti-His_6 antibody. Polyubiquitinated (Poly-Ub) Cdon, unmodified full-length (FL) Cdon, and Poly-Ub-ZSWIM8 are indicated on the right side. The membranes were cut prior to hybridization with antibodies. Full-length blots are presented in Supplementary Figure 12.
