# Supplementary material for: ZSWIM8 is a myogenic protein that partly prevents C2C12 differentiation
Source: Sci Rep. 2021 Oct 22;11:20880. doi: 10.1038/s41598-021-00306-6 (PMC8536758; doi:10.1038/s41598-021-00306-6)
Supplement: Supplementary file 5 — Supplementary Information 5. [file 41598_2021_306_MOESM5_ESM.pdf]

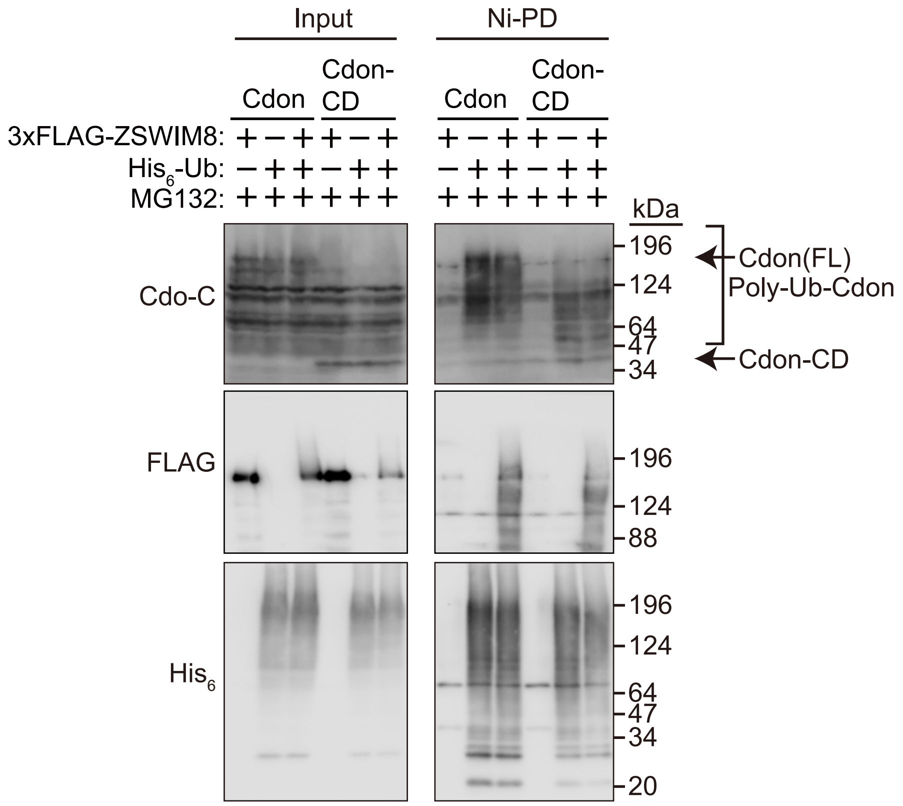

Supplementary Figure 5. Overexpression of ZSWIM8 does not induce ubiquitination of Cdon cytoplasmic fragment

His_6-ubiquitination of cytoplasmic region of Cdon is not dependent on ZSWIM8 overexpression. Un-tagged full-length Cdon or the cytoplasmic region of Cdon (Cdon-CD), 3×FLAG-ZSWIM8, and His_6-ubiquitin (Ub) were expressed in HEK293T cells with the indicated combinations. Ni-PD and immunoblot analysis were performed as in Supplementary Figure 1, except with an anti-HA antibody. The anti-Cdon-C antibody was used to detect both full-length Cdon and Cdon-CD. Polyubiquitinated (Poly-Ub) and unmodified full-length (FL) Cdon or Cdon-CD are indicated on the right side. The membranes were cut prior to hybridization with antibodies. Full-length blots are presented in Supplementary Figure 13.
