# Supplementary material for: ZSWIM8 is a myogenic protein that partly prevents C2C12 differentiation
Source: Sci Rep. 2021 Oct 22;11:20880. doi: 10.1038/s41598-021-00306-6 (PMC8536758; doi:10.1038/s41598-021-00306-6)
Supplement: Supplementary file 6 — Supplementary Information 6. [file 41598_2021_306_MOESM6_ESM.pdf]

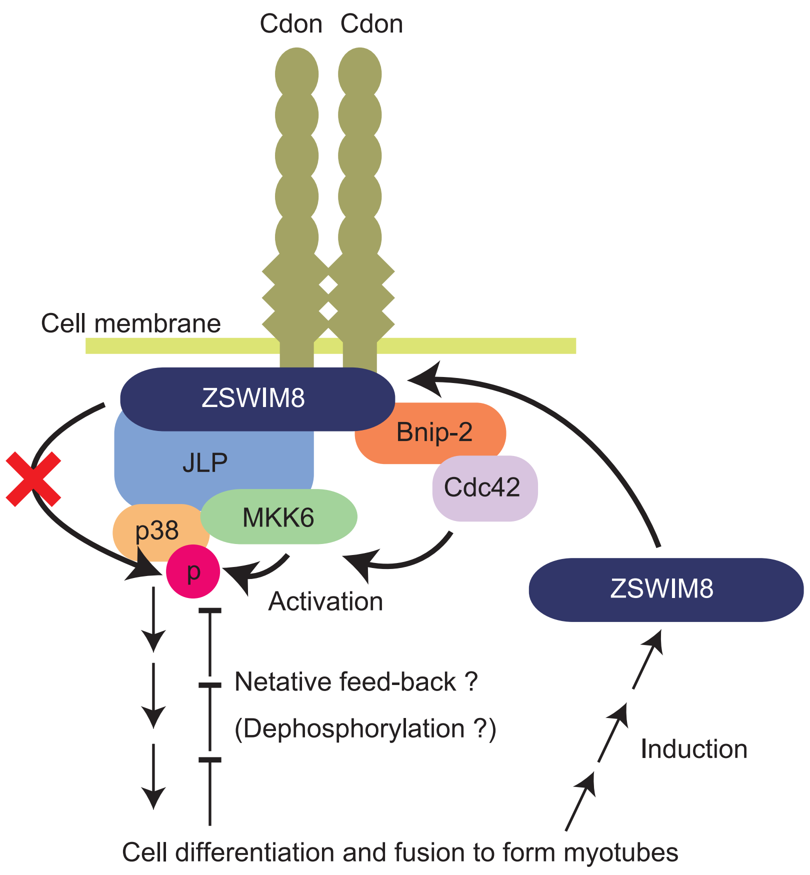

Supplementary Figure 6. Hypothetical model of ZSWIM8 and Cdon complex-mediated signaling

This hypothetical model shows the ZSWIM8 and Cdon complex-mediated signaling. C2C12 cell differentiation and fusion to form myotubes that induce ZSWIM8 expression. Cdon forms a complex with Bnip-2, Cdc42, JLP, MKK6, p38α/β MAPK, and ZSWIM8. Cdc42 activates MKK6, which in turn phosphorylates p38α/β MAPK. ZSWIM8 does not affect p38α/β MAPK activation. Activated p38α/β MAPK binds to a large number of active promoters and phosphorylates a variety of substrates during C2C12 differentiation to regulate myogenic gene expression. C2C12 differentiation may negatively regulate p38α/β MAPK via several pathways in ZSWIM8 knockdown cells. The model was drawn using Adobe Illustrator CS6. https://www.adobe.com/
