# Supplementary figures and images for: ZSWIM8 is a myogenic protein that partly prevents C2C12 differentiation
Source: Sci Rep. 2021 Oct 22;11:20880. doi: 10.1038/s41598-021-00306-6 (PMC8536758; doi:10.1038/s41598-021-00306-6)

Figure 1A

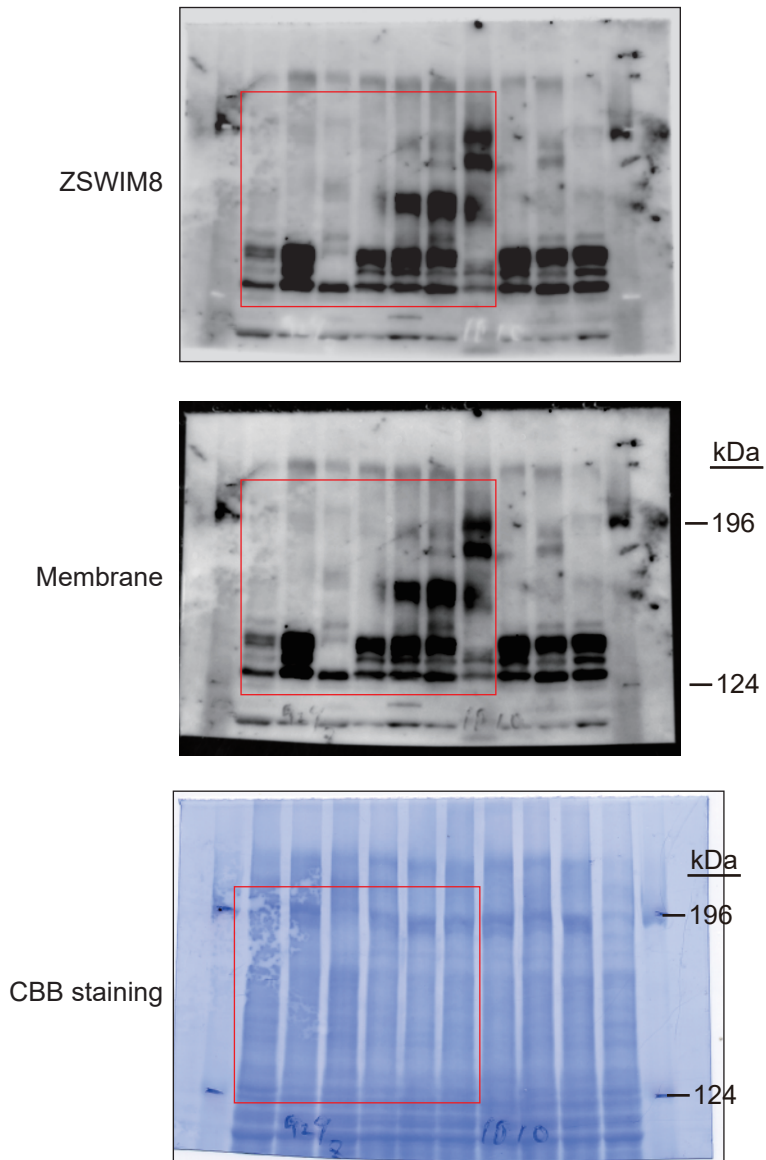

Figure 1B

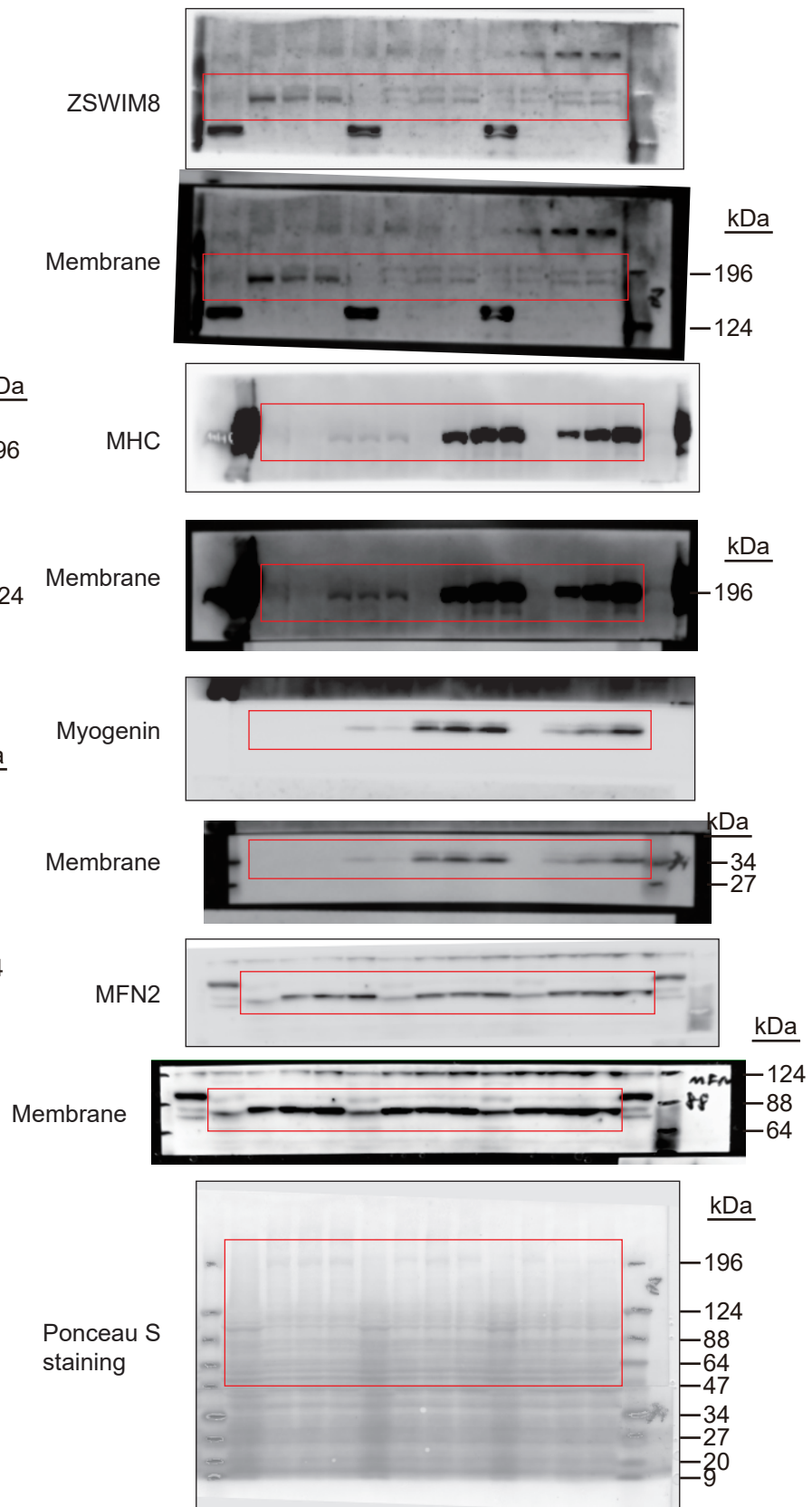

Supplementary Figure 7. Uncropped images of Figure 1.

Supplement: Supplementary file 7 — Supplementary Information 7. [file 41598_2021_306_MOESM7_ESM.pdf]

Figure 3B

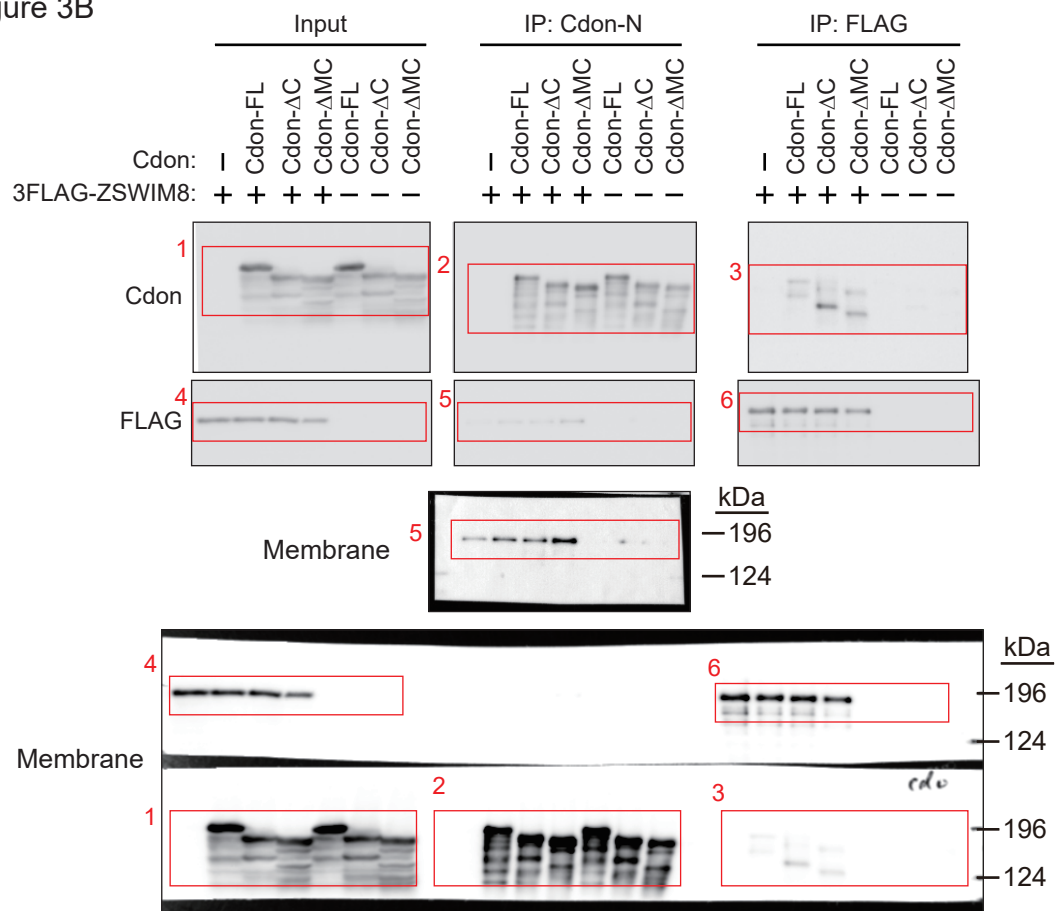

Figure 3C

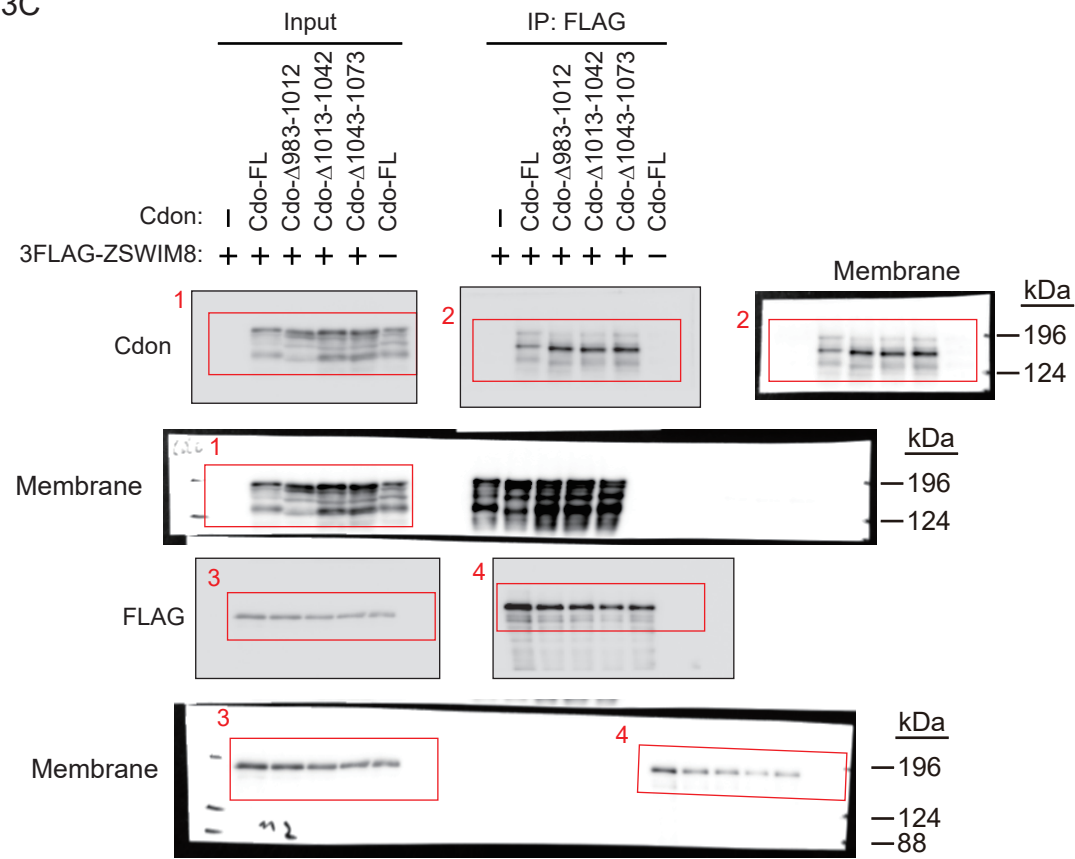

Supplementary Figure 8. Uncropped images of Figure 3.

Supplement: Supplementary file 8 — Supplementary Information 8. [file 41598_2021_306_MOESM8_ESM.pdf]

Figure 5A

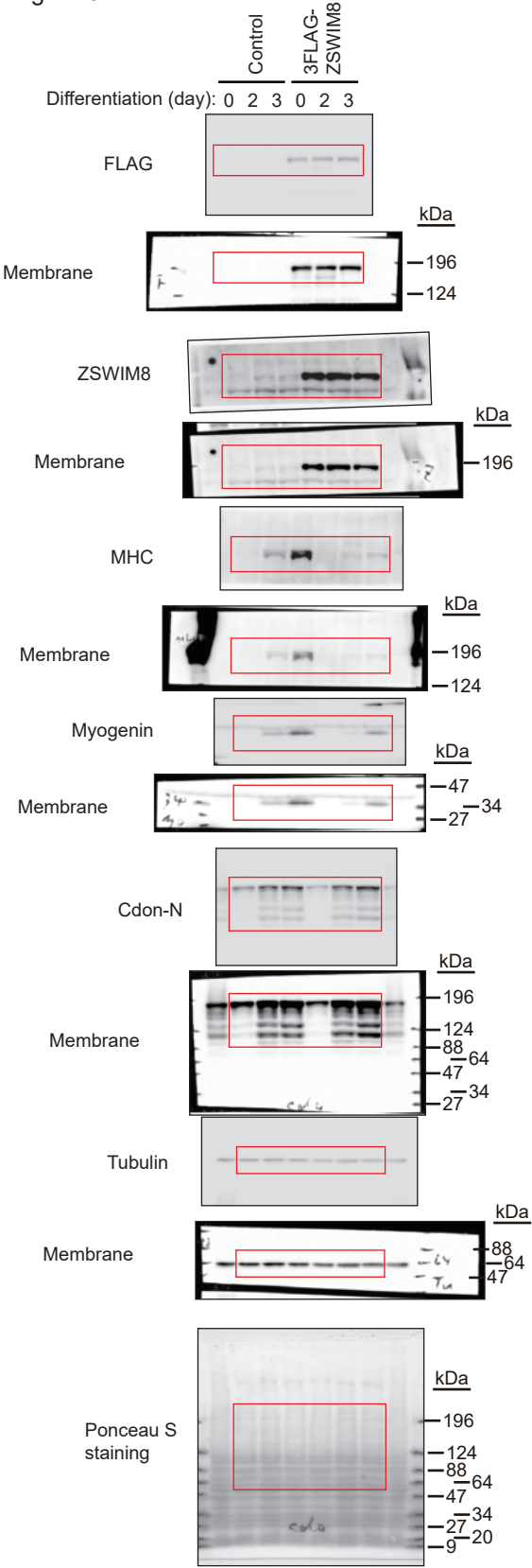

Figure 5C

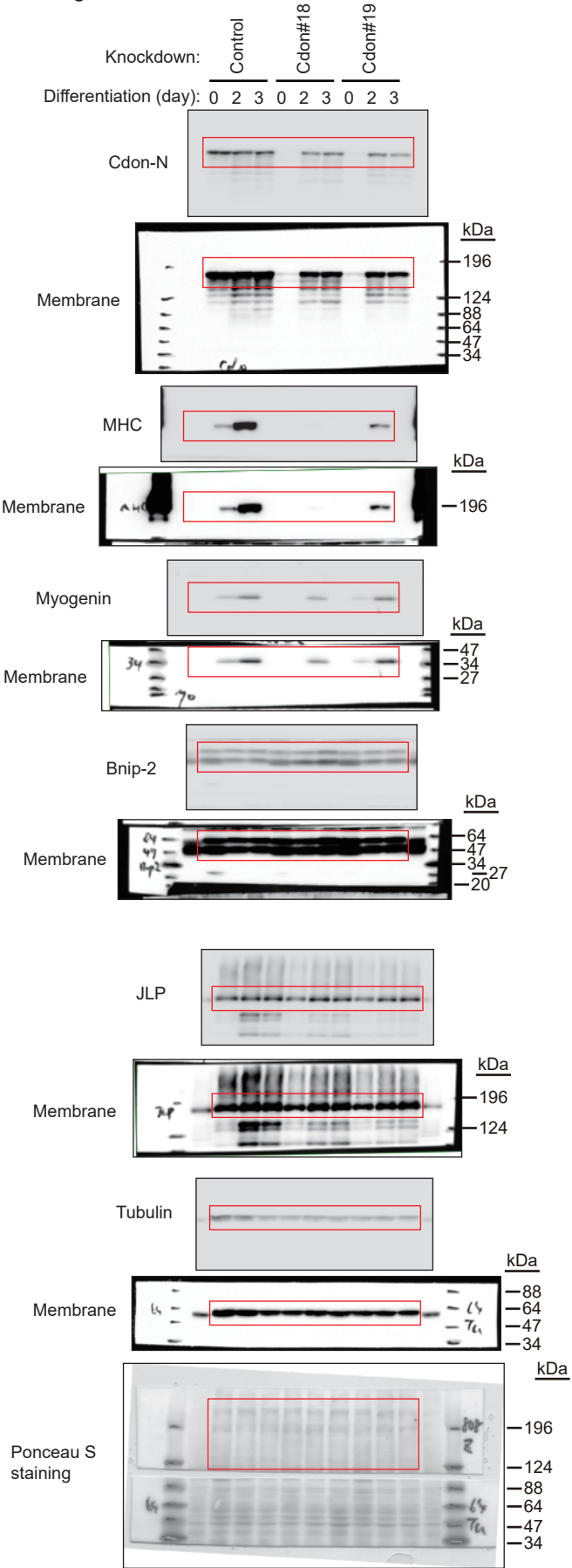

Supplementary Figure 9. Uncropped images of Figure 5.

Supplement: Supplementary file 9 — Supplementary Information 9. [file 41598_2021_306_MOESM9_ESM.pdf]

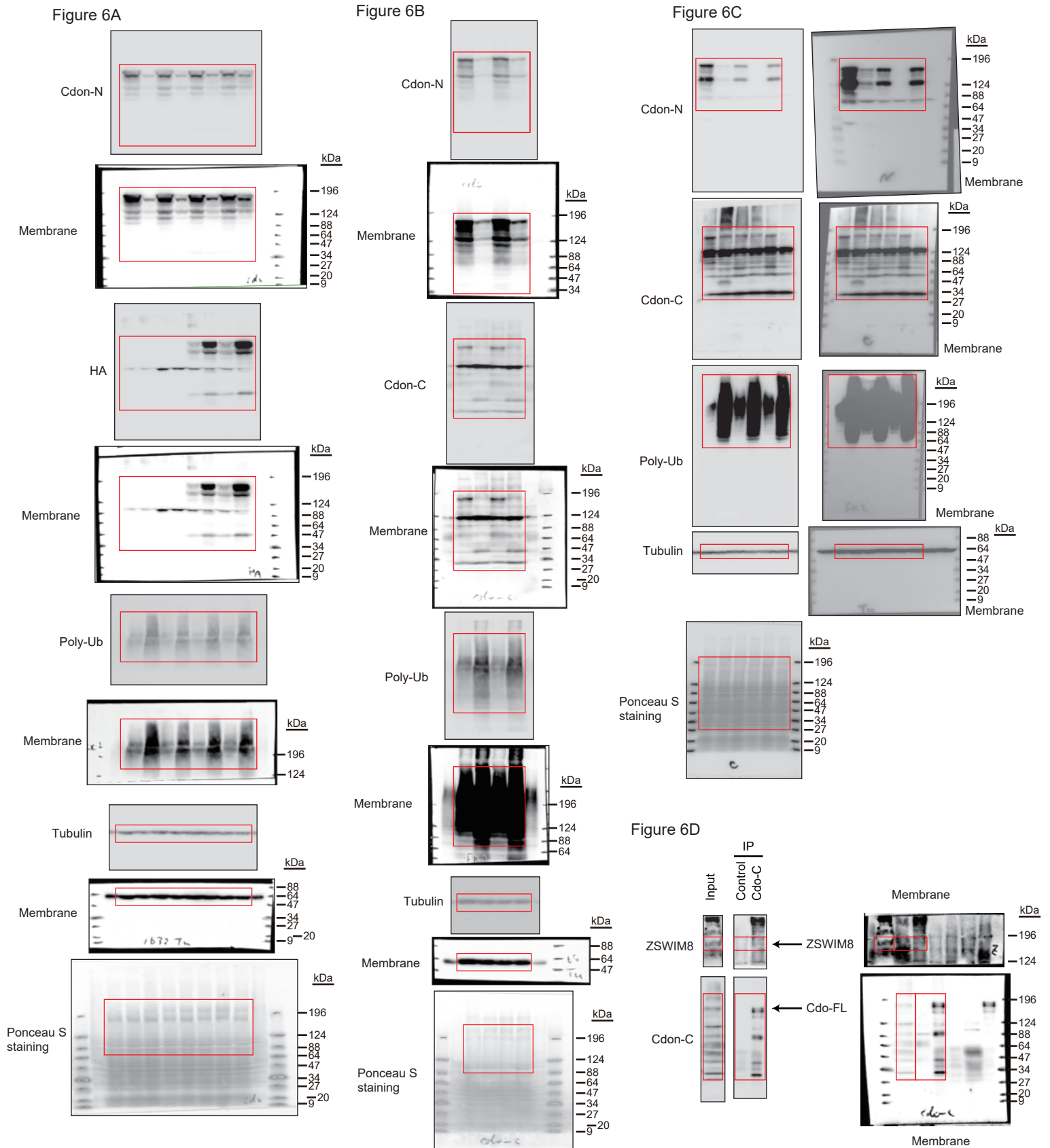

Supplementary Figure 10. Uncropped images of Figure 6.

Supplement: Supplementary file 10 — Supplementary Information 10. [file 41598_2021_306_MOESM10_ESM.pdf]

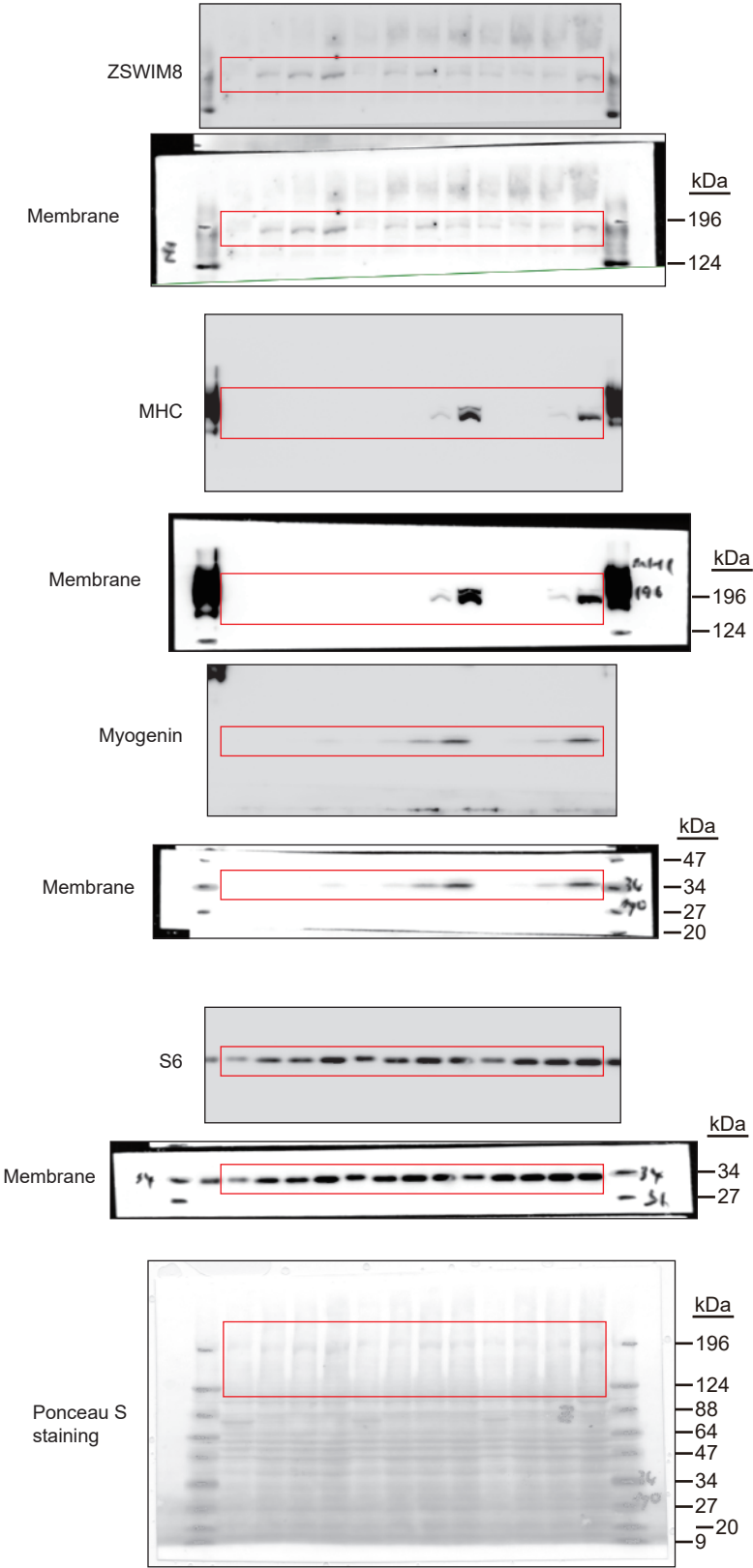

Supplementary Figure 12. Uncropped images of Supplementary Figure 1.

Supplement: Supplementary file 12 — Supplementary Information 12. [file 41598_2021_306_MOESM12_ESM.pdf]

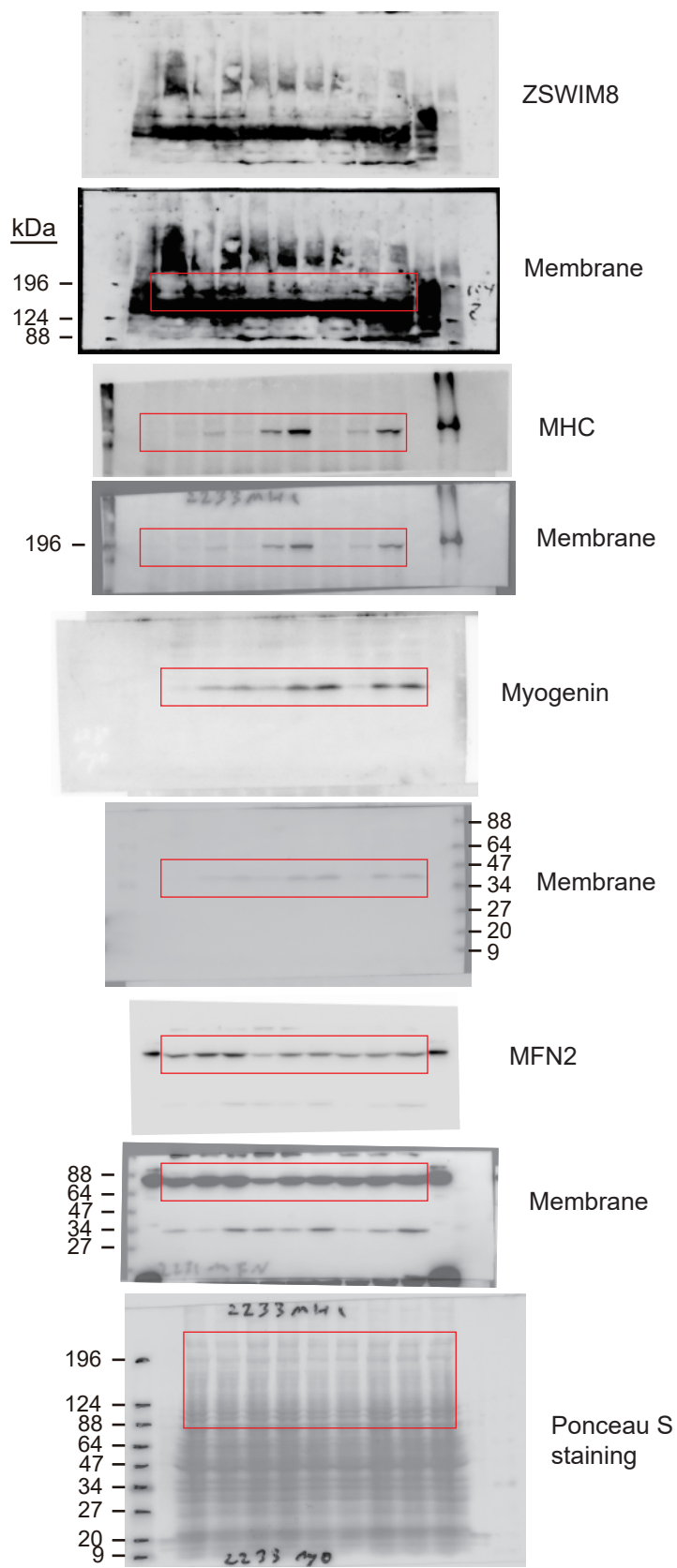

Supplementary Figure 13. Uncropped images of Supplementary Figure 2.

Supplement: Supplementary file 13 — Supplementary Information 13. [file 41598_2021_306_MOESM13_ESM.pdf]

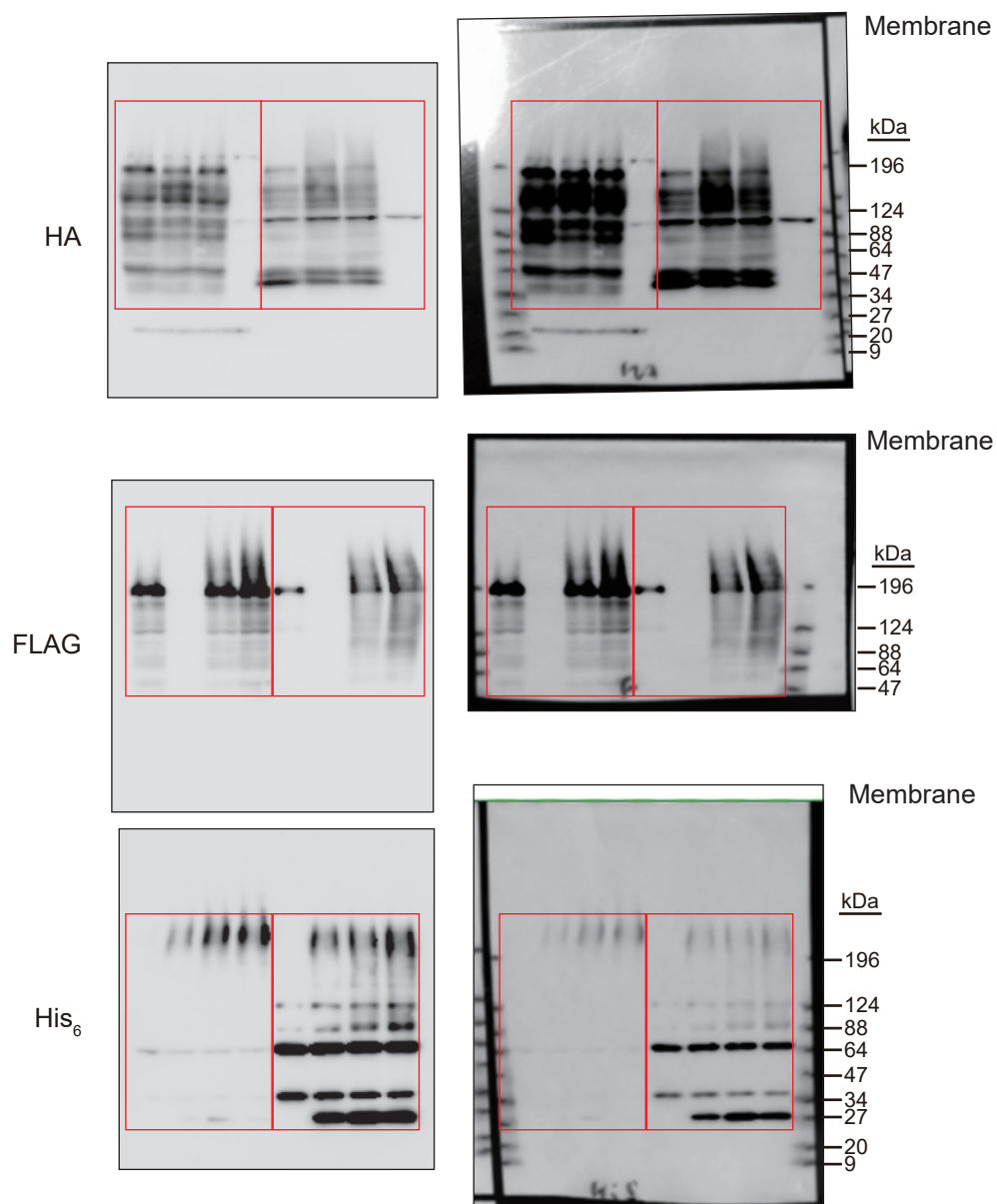

Supplementary Figure 14. Uncropped images of Supplementary Figure 4.

Supplement: Supplementary file 14 — Supplementary Information 14. [file 41598_2021_306_MOESM14_ESM.pdf]

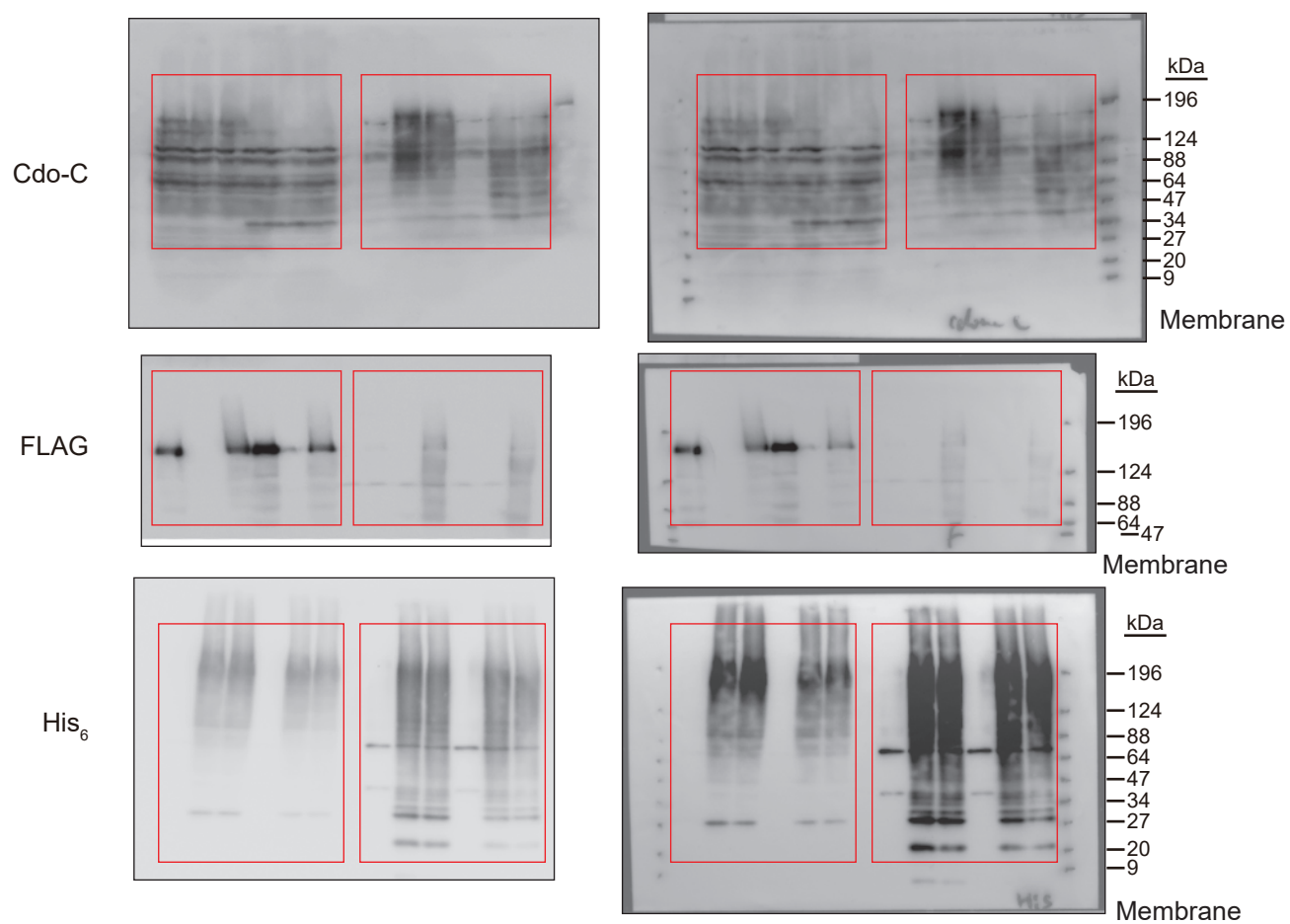

**Supplementary Figure 15. Uncropped images of Supplementary Figure 5.**

Supplement: Supplementary file 15 — Supplementary Information 15. [file 41598_2021_306_MOESM15_ESM.pdf]
