# Supplementary material for: ZSWIM8 is a myogenic protein that partly prevents C2C12 differentiation
Source: Sci Rep. 2021 Oct 22;11:20880. doi: 10.1038/s41598-021-00306-6 (PMC8536758; doi:10.1038/s41598-021-00306-6)
Supplement: Supplementary file 11 — Supplementary Information 11. [file 41598_2021_306_MOESM11_ESM.pdf]

Supplementary Figure 11_Okumura

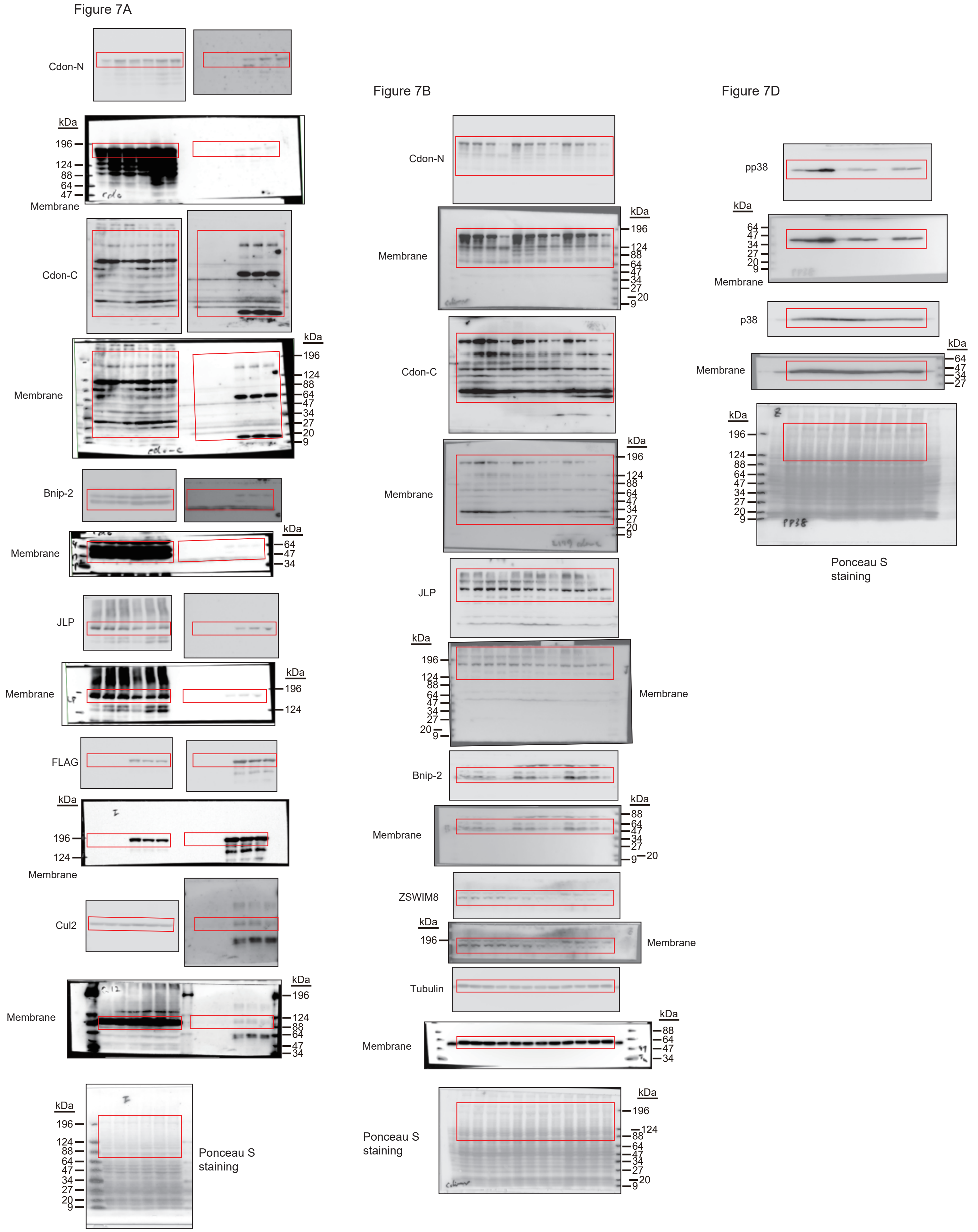

Supplementary Figure 11. Uncropped images of Figure 7.
